# *Selaginella doederleinii*-derived extracellular vesicle-like particles suppress lung cancer with ferroptosis-associated changes and modulation of the FABP4/PPARG/GPX4 axis

**DOI:** 10.3389/fonc.2026.1829211

**Published:** 2026-05-29

**Authors:** Hongyao Chen, Jingting Zhang, Ling Wu, Zhibing Wang, Wei Peng, Zuomei He, Xiaoning Tan, Renyi Yang, Puhua Zeng

**Affiliations:** 1 Hunan Provincial Hospital of Integrated Traditional Chinese and Western Medicine, Hunan University of Chinese Medicine, Changsha, China; 2 Institute of Traditional Chinese Medicine Oncology, Hunan Academy of Chinese Medicine, Changsha, China

**Keywords:** ferroptosis, lung cancer, plant-derived extracellular vesicles, *Selaginella doederleinii*, Traditional Chinese Medicine

## Abstract

**Introduction:**

*Selaginella doederleinii*, a traditional Chinese medicinal herb, has reported antitumor activity; however, the instability and batch-to-batch variability of crude preparations hinder translational development. Plant-derived exosome-like nanovesicles may serve as a pharmaceutical platform for multicomponent cargo delivery and potentially improve bioavailability. This study aimed to determine whether *Selaginella doederleinii*–derived extracellular vesicle-like particles (SD-EVLPs) suppress lung cancer and to explore the underlying mechanisms.

**Methods:**

SD-EVLPs were purified by differential ultracentrifugation and sucrose density–gradient separation, and characterized by nanoparticle tracking analysis and transmission electron microscopy (TEM). LC–MS cargo profiling and bioinformatic analyses were used to prioritize ferroptosis-related targets and pathways. Cellular uptake and antitumor activities were assessed in lung cancer cells using proliferation, colony formation, migration, and invasion assays. Ferroptosis-associated alterations were evaluated by measuring Fe^2+^, ROS, lipid peroxidation/MDA, and GSH, together with RT-qPCR and Western blot analyses of key regulators. Antitumor efficacy, tumor ferroptosis-related indices/molecular markers, and safety were further examined in nude-mouse xenografts.

**Results:**

SD-EVLPs were nanosized vesicles carrying multiple putative bioactive components. Bioinformatics suggested enrichment of ferroptosis-related signals in the PPAR pathway and implicated a candidate FABP4/PPARG/GPX4 axis. SD-EVLPs were efficiently internalized and dose-dependently inhibited proliferation, migration, and invasion, accompanied by increased Fe^2+^ and oxidative stress, enhanced lipid peroxidation, GSH depletion, and modulation of the ferroptosis defense machinery. *In vivo*, SD-EVLPs suppressed tumor growth and reduced proliferative markers, with ferroptosis-related changes in tumor tissues consistent with *in vitro* trends and no overt hepatorenal toxicity.

**Discussion:**

These findings suggest that SD-EVLPs exhibit anti–lung cancer activity, which may be associated with ferroptosis-related changes and modulation of the FABP4/PPARG/GPX4-associated pathway. SD-EVLPs may represent a potential plant-derived nanovesicle platform for lung cancer intervention.

## Graphical Abstract

**Figure:**
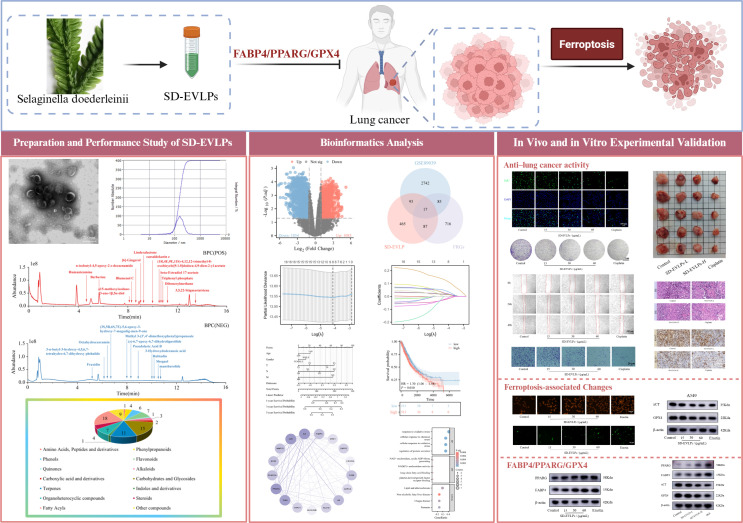
GRAPHICAL ABSTRACT

## 1 Background

Lung cancer is one of the malignancies with the highest incidence and mortality worldwide. Driven by factors such as smoking, environmental pollution, and occupational exposure, the incidence of lung cancer continues to rise. Global cancer epidemiology shows that in 2022 there were approximately 2.5 million new lung cancer cases and about 1.8 million new deaths from lung cancer, accounting for 12.4% of all newly diagnosed malignancies and 18.7% of cancer deaths, respectively (1), posing a serious threat to human health. In recent years, notable advances have been made in lung cancer diagnosis and treatment, including surgery, chemotherapy, radiotherapy, targeted therapy, and immunotherapy; however, the overall survival of patients remains unsatisfactory, and lung cancer prevention and control still face severe challenges. In addition, these therapies are often accompanied by adverse effects, and problems such as drug resistance and limited treatment options persist, leading to unfavorable outcomes for many patients. In recent years, ferroptosis, as a novel form of regulated cell death, has gradually attracted increasing attention, and its core features are the iron-dependent accumulation of lipid peroxidation and an imbalance in antioxidant defense (2). Studies have shown that lung cancer is often accompanied by dysregulated iron homeostasis and elevated redox levels, thereby conferring a ferroptosis sensitivity that can be therapeutically exploited (3). Meanwhile, ferroptosis is closely related to lung cancer initiation, progression, and treatment response, and has been considered a potential strategy to enhance efficacy and overcome drug resistance (4). In particular, in therapy-tolerant/persister cell states, cells exhibit dependence on the GPX4-mediated lipid peroxide repair pathway, and therefore show marked vulnerability to ferroptosis triggered by GPX4 inhibition (5, 6). Accordingly, developing interventions targeting ferroptosis may provide a new entry point for overcoming resistance and improving therapeutic efficacy.

*Selaginella doederleinii* Hieron., a traditional Chinese medicinal herb, has been widely used in clinical practice for the treatment of malignant tumors such as nasopharyngeal carcinoma, lung cancer, liver cancer, cervical cancer, and ovarian cancer. Modern pharmacological studies have found that *Selaginella doederleinii* possesses antioxidant and anti-inflammatory activities, can regulate tumor cell metabolism, and can inhibit tumor growth and metastasis, further enriching our understanding of its antitumor effects. However, from a translational perspective, as a fresh herbal medicine, *Selaginella doederleinii* is often constrained by difficulties in preservation, batch-to-batch inconsistency, and insufficient preparation stability, which can affect long-term stable supply and controllable exposure to its active constituents.

With the development of life science and technology, extracellular vesicles (EVs) have attracted considerable attention for their therapeutic potential in diseases. EVs are nanoscale lipid bilayer vesicles actively secreted by cells. They can carry bioactive molecules such as DNA, RNA, proteins, lipids, and metabolites, and by delivering their cargos to recipient cells, they regulate gene expression and cellular functions. Through these processes, EVs participate in key biological events including immune regulation, tissue repair, angiogenesis, and tumor metastasis (7). Plant-derived extracellular vesicles (PDEVs) are nanoscale lipid bilayer vesicles secreted by plant cells and are widely present in plant sap, extracellular fluids, and tissue culture media. PDEVs are released in a non-cell-autonomous manner, transport bioactive molecules across species, and exhibit unique biological functions and application potential. In recent years, PDEVs have attracted increasing interest in anticancer and immunomodulatory fields due to their abundant sources, favorable bioactivity, biocompatibility, low immunogenicity, and safety advantages, and their therapeutic potential has been increasingly recognized (8).

PDEVs extracted from fresh medicinal plants carry various lipids, proteins, and mRNA from plant cells, as well as the active constituents of the fresh herbs themselves. Their isolation can be relatively straightforward, which may facilitate scalable preparation and enrichment. Moreover, because they are typically prepared without high-temperature inactivation, PDEVs may retain the intrinsic medicinal activity of fresh herbs to a considerable extent. Their lipid structure may support intestinal uptake and enable the encapsulation or loading of poorly water-soluble small-molecule substances from fresh herbs (9, 10). Collectively, these features suggest that fresh-herb PDEV technology may offer a promising approach to addressing the preservation limitations of fresh herbal medicines and may contribute to disease intervention. In this context, delivery of the active components of *Selaginella doederleinii* via PDEVs may confer improved bioavailability and therapeutic advantages, providing a potential new avenue for its application in lung cancer treatment.

Based on this, we isolated *Selaginella doederleinii*–derived extracellular vesicle-like particles (SD-EVLPs), systematically evaluated their inhibitory effects on lung cancer through bioinformatics combined with *in vitro* and *in vivo* experiments, and further integrated ferroptosis-related phenotyping with analyses of candidate molecular pathways to explore whether SD-EVLPs are associated with ferroptosis-related responses in lung cancer cells, thereby providing experimental support for the development of novel anti-lung-cancer strategies.

## 2 Methods

### 2.1 Cell lines and animals

Human lung cancer cell lines A549 (Cat. No.: CL-0016) and NCI-H1299 (Cat. No.: CL-0165) were purchased from Wuhan Procell Life Science & Technology Co., Ltd. Four-week-old male BALB/c nude mice (SPF grade) were purchased from Hunan Silaike Jingda Laboratory Animal Co., Ltd. (Changsha, China), with the Animal Production License No.: SCXK (Xiang) 2021-0002. The mice were housed in the Animal Experiment Center of Hunan University of Chinese Medicine under standard conditions (temperature 24–25 °C, relative humidity 50–60%, and a 12-hour light/dark cycle), with free access to food and water. All experimental protocols were approved by the Animal Experimental Ethics Committee of Hunan University of Chinese Medicine (approval number: LL2022060804).

### 2.2 Drugs and reagents

*Selaginella doederleinii* was purchased from Bozhou Qihongtang Pharmaceutical Co., Ltd. and identified by Zuhan Xiao, a traditional Chinese medicine pharmacist at the Affiliated Hospital of Hunan Academy of Chinese Medicine, as the whole herb of *Selaginella doederleinii* Hieron. (Selaginellaceae, Selaginella). Cisplatin (Cat. No.: ST1164-50mg), Erastin (Cat. No.: SC0224-5mg), and Imidazole Ketone Erastin (IKE) (Cat. No.: Y169346-5mg) were purchased from Shanghai Beyotime Biotechnology Co., Ltd.

PBS buffer (Cat. No.: PB180521), A549 cell culture medium (Cat. No.: CM-0016), NCI-H1299 cell culture medium (Cat. No.: CM-0165), and 0.25% trypsin solution (Cat. No.: PB180228) were purchased from Wuhan Procell Life Science & Technology Co., Ltd. 0.45 μm sterile filters (Cat. No.: 5191-4287) were obtained from Shanghai Xiyan Scientific Instruments Co., Ltd. Sucrose (Cat. No.: 15700101) was purchased from Xilong Scientific Co., Ltd. Quick-seal centrifuge tubes (Cat. No.: 342413) were purchased from Beckman Coulter, Inc. BCA protein assay kit (Cat. No.: E-BC-K318-M) was purchased from Wuhan Elabscience Biotechnology Co., Ltd. Cell membrane staining reagent DiI (Cat. No.: BB-441919), DAPI staining reagent (Cat. No.: BB-4401), and CCK-8 stop solution (Cat. No.: BB-4281) were purchased from Nanjing Fengfeng Biomedical Technology Co., Ltd. EdU-488 Cell Proliferation Assay Kit (Cat. No.: C0071S), Transwell 24-well chamber plates (8 μm pore size, PC membrane) (Cat. No.: FTW067-12Ins), Malondialdehyde (MDA) assay kit (Cat. No.: S0131M), and Reactive Oxygen Species (ROS) assay kit (Cat. No.: S0033M) were purchased from Shanghai Beyotime Biotechnology Co., Ltd. FerroOrange ferrous ion detection probe (Cat. No.: M42835) was purchased from AoMei Biotechnology (Shanghai) Co., Ltd. Reduced Glutathione (GSH) content assay kit (Cat. No.: BC1175) was purchased from Beijing Aomi Jiade Pharmaceutical Technology Co., Ltd. Anti-E-cadherin (Cat. No.: 20874-1-AP), anti-N-cadherin (Cat. No.: 22018-1-AP), anti-GPX4 (Cat. No.: 67763-1-IG), anti-xCT (Cat. No.: 26864-1-AP), anti-FABP4 (Cat. No.: 12802-1-AP), anti-PPARG (Cat. No.: 16643-1-AP), HRP-conjugated goat anti-rabbit secondary antibody (Cat. No.: SA00001-2), and HRP-conjugated goat anti-mouse secondary antibody (Cat. No.: SA00001-1) were purchased from Wuhan Sanying Biotechnology Co., Ltd.

### 2.3 Instruments

An ultra-high-speed centrifuge (Optima XE series, Beckman Coulter, Inc., USA); ZetaView PMX 110 nanoparticle tracking analyzer (Particle Metrix GmbH, Germany); Enspire multifunctional microplate reader (PerkinElmer, Inc., USA); HT7700 transmission electron microscope (Hitachi, Ltd., Japan); BCA protein assay kit (Lot No.: E-BC-K318-M; Wuhan Elabscience Biotechnology Co., Ltd.); Mini-PROTEAN Tetra vertical electrophoresis system and ChemiDoc XRS+ Imager chemiluminescence system (Bio-Rad Laboratories, Inc., USA).

### 2.4 Preparation and characterization of SD-EVLPs

#### 2.4.1 Extraction of SD-EVLPs using differential centrifugation and ultracentrifugation

Fresh *Selaginella doederleinii* was washed, cut into small pieces, and homogenized in a suitable volume of pre-cooled (4°C) PBS using a homogenizer. The resulting juice was centrifuged at 1,000 ×g for 30 min at 4°C, and the supernatant was collected and filtered using a diaphragm booster pump. The filtrate was centrifuged at 10,000 ×g for 60 min, followed by filtration through a 0.45 μm sterile filter. The filtrate was transferred to new centrifuge tubes and ultracentrifuged at 100,000 ×g for 90 min at 4°C. The supernatant was discarded, and the pellet in each tube was resuspended in 1 mL PBS to obtain the crude SD-EVLPs suspension.

#### 2.4.2 Purification of SD-EVLPs

Purification was performed using sucrose density gradient ultracentrifugation. Sucrose solutions of 8%, 30%, 45%, and 60% (w/v) were prepared in ultrapure water. The SD-EVLPs sample was placed in a centrifuge tube, and sucrose solutions of increasing concentrations were carefully layered beneath the sample. The tube was centrifuged at 110,000 ×g for 90 min. The SD-EVLPs-containing band was gently collected, diluted with PBS to the full tube volume, and centrifuged again at 100,000 ×g for 60 min to remove residual sucrose. The pellet was resuspended in PBS and aliquoted before being stored at −80°C until use, and repeated freeze–thaw cycles were avoided.

#### 2.4.3 Transmission electron microscopy for morphological observation of SD-EVLPs

SD-EVLPs suspensions were mixed with electron microscopy fixative at a 1:10 ratio to prepare the samples. Copper grids were placed on filter paper, and 10 μL of the sample was applied onto each grid. After standing for 3 min, excess liquid was gently removed from the edge of the grid using filter paper. One drop of PBS was then added to rinse the grid, followed by air-drying at room temperature. Negative staining was performed with 3% phosphotungstic acid at room temperature for 5 min, after which the grids were dried again. The morphology of SD-EVLPs was subsequently examined and imaged using a transmission electron microscope operated at an accelerating voltage of 80–120 kV.

#### 2.4.4 Nanoparticle tracking analysis for particle size and concentration

NTA was performed using a ZetaView PMX 110 nanoparticle tracking analyzer (Particle Metrix, Germany) equipped with the associated ZetaView software (version 8.04.02 SP2). SD-EVLPs suspensions were diluted with PBS at 25°C to reach the optimal particle concentration recommended by the instrument. One milliliter of the diluted sample was equilibrated at room temperature for 2 min before loading into the measurement chamber. NTA measurement was recorded and analyzed at 11 pre-defined positions across the chamber. The ZetaView system was calibrated using 100 nm polystyrene particles prior to measurement, and all analyses were conducted with the operating temperature maintained at approximately 25°C.

#### 2.4.5 Determination of protein concentration using the BCA assay

A 2 mg/mL protein standard was serially diluted to concentrations of 0, 0.03125, 0.0625, 0.125, 0.25, 0.5, 1, and 2 mg/mL. Reagents A and B from the kit were mixed at a 50:1 ratio and kept protected from light. SD-EVLPs suspensions were diluted 10-fold. Twenty microliters of each protein standard and diluted SD-EVLPs sample were added into wells of a 96-well plate. The plate was wrapped in aluminum foil and incubated at 37°C for 30 min. Protein concentrations were measured at 562 nm using a microplate reader.

#### 2.4.6 Liquid chromatography–mass spectrometry analysis of active components in SD-EVLPs

SD-EVLPs samples were homogenized, and 100 μL aliquots were transferred into 1.5 mL centrifuge tubes. A total of 900 μL of 70% methanol (containing mixed internal standards, 4 μg/mL) was added, followed by vortexing for 1 min. Samples were ultrasonically extracted in an ice-water bath for 60 min and centrifuged at 12,000 rpm for 10 min at 4°C. Then, 200 μL of the supernatant was transferred into LC-MS vials for analysis. LC-MS analysis was performed using an ACQUITY UPLC I-Class HF system coupled to a QE high-resolution mass spectrometer. Chromatographic conditions: ACQUITY UPLC HSS T3 column (100 mm × 2.1 mm, 1.8 μm); column temperature 45°C; mobile phase A: water with 0.1% formic acid; mobile phase B: acetonitrile; flow rate 0.35 mL/min; injection volume 2 μL. PDA scanning range was set to 210–400 nm. Mass spectrometry was conducted using a HESI ion source in both positive and negative modes. Data acquisition mode was DDA with Full MS/dd-MS² (TOP 8) scanning.

Raw data were processed using Progenesis QI v3.0 for baseline filtering, peak identification, integration, retention time correction, alignment, and normalization. Compound identification was performed based on accurate mass, MS/MS fragmentation patterns, and isotopic distribution using the TCM compound database. Components with a total identification score ≥ 40 were retained. After merging and deduplicating positive- and negative-ion results, relative peak area percentages were normalized to a total of 100% to obtain qualitative and quantitative profiles.

#### 2.4.7 Screening of active components in SD-EVLPs

Active components identified by LC-MS were screened using the Traditional Chinese Medicine Systems Pharmacology Database and Analysis Platform (TCMSP). Compounds with OB ≥ 30% and DL ≥ 0.18 were considered bioactive (11). Additionally, SwissADME predictions were used to assess gastrointestinal (GI) absorption and drug-likeness. Compounds with “High” GI absorption and at least two “Yes” results among drug-likeness parameters were included. The union of results from both platforms was taken as the final set of SD-EVLP active components.

### 2.5 Bioinformatics analysis

#### 2.5.1 Identification of SD-EVLPs intervention targets and their expression patterns in lung cancer patients

Based on the 25 active components of SD-EVLPs identified and screened via LC-MS analysis in Sections 2.4.6 and 2.4.7, the corresponding three-dimensional structures (SMILES or SDF format) were retrieved from the PubChem database (https://pubchem.ncbi.nlm.nih.gov/). Potential drug targets of these active compounds were predicted using the SwissTargetPrediction database (http://www.swisstargetprediction.ch/). After removing duplicate entries, a non-redundant set of putative SD-EVLPs intervention targets was obtained. Ferroptosis-related genes (FRGs) were downloaded from the FerrDb database (http://www.zhounan.org/ferrdb) and integrated with an early-preview collection of ferroptosis-related publications indexed in PubMed in 2022, resulting in an expanded FRG dataset. In the Gene Expression Omnibus (GEO) database (https://www.ncbi.nlm.nih.gov/geo/), the lung cancer expression dataset GSE89039 was retrieved using “Lung Cancer” as the keyword. Following log2 transformation and normalization, box plots and principal component analysis (PCA) were conducted to assess normalization quality and sample distribution. Differential expression analysis of the normalized GSE89039 transcriptome dataset was performed using the “limma” package in R. Differentially expressed genes (DEGs) in lung cancer were identified using |logFC| > 1 and *P* < 0.05 as the significance thresholds. Overlap analysis among SD-EVLP targets, FRGs, and lung cancer differentially expressed genes was performed to identify potential SD-EVLP targets involved in ferroptosis regulation in lung cancer.

For external validation, RNA-seq data (STAR pipeline) from the TCGA-LUAD and TCGA-LUSC projects, including 1,149 lung cancer tissues and 108 adjacent normal tissues, were obtained from The Cancer Genome Atlas (TCGA) database (https://portal.gdc.cancer.gov/). Paired and unpaired t-tests were performed to compare expression differences between tumor and normal tissues. Protein-level validation was further conducted using immunohistochemistry data for the identified targets from the Human Protein Atlas (HPA) database (https://www.proteinatlas.org/).

#### 2.5.2 Prognostic analysis of SD-EVLPs intervention targets in lung cancer patients

Based on the RNA-seq data and clinical information from the TCGA-LUAD and TCGA-LUSC cohorts, LASSO regression with 10-fold cross-validation was performed to identify prognosis-related candidate genes among the ferroptosis-related SD-EVLP intervention targets. The selected genes were further analyzed by univariate and multivariate Cox regression to construct a combined risk score. Patients were stratified into high- and low-risk groups according to the median risk score, and survival differences were evaluated by Kaplan–Meier analysis and log-rank testing.

Clinical variables, including age, sex, TNM stage, and risk score, were incorporated into Cox regression analyses to assess their independent prognostic significance. A nomogram integrating the risk score and clinical characteristics was constructed for supportive internal evaluation, and calibration curves were used to assess agreement between predicted and observed 1-, 3-, and 5-year overall survival.

#### 2.5.3 Network pharmacology analysis, molecular docking, and molecular dynamics simulation

A Protein–Protein Interaction (PPI) network of SD-EVLPs intervention targets was constructed using the STRING database (https://cn.string-db.org/) and visualized in Cytoscape 3.9.1. Gene Ontology (GO) and Kyoto Encyclopedia of Genes and Genomes (KEGG) enrichment analyses of SD-EVLPs intervention targets were performed using the “clusterProfiler” and “org.Hs.eg.db” packages in R to identify candidate pathways related to ferroptosis. The enrichment results were visualized as bubble plots.

Molecular docking was conducted to evaluate the binding potential between SD-EVLPs active components and candidate target proteins. The three-dimensional SDF structures of the 25 active compounds were obtained from the PubChem database (https://pubchem.ncbi.nlm.nih.gov/). X-ray crystallographic structures of target proteins were downloaded from the RCSB Protein Data Bank (PDB) (http://www.rcsb.org/) (12) and saved in standard PDB format. Docking simulations were performed using AutoDock 4.2.6 (13). Based on the crystallographic coordinates, active binding pockets were precisely defined using the grid-box module in AutoDock Tools. After optimization of docking parameters, the Lamarckian genetic algorithm was employed to compute docking conformations. Protein–ligand interaction patterns were analyzed using the Protein–Ligand Interaction Profiler (PLIP) platform (14) and visualized in PyMOL v2.6 (15). Binding free energy, ligand–receptor interaction modes, and key interacting amino acid residues were used for result evaluation. MD simulation was performed to assess the stability of protein–ligand complexes with high docking scores and to elucidate their conformational dynamics at the atomic level. MD simulations were carried out using the GROMACS 2025.2 software package (16). Each complex was placed in a periodic cubic simulation box, solvated with the TIP3P water model, and parameterized using the CHARMM36 force field. Sodium and chloride ions were added to achieve a physiological salt concentration (150 mM NaCl). After energy minimization, NVT and NPT equilibration phases (each 100 ps) were performed with positional restraints applied to heavy atoms, followed by a 100 ns production run with a 2 fs time step. Trajectories were processed to remove periodic boundary artifacts and recentered for analysis. To evaluate the structural stability of the complexes, several parameters were calculated, including root mean square deviation (RMSD) of Cα atoms, radius of gyration (Rg), root mean square fluctuation (RMSF) of residues, solvent-accessible surface area (SASA), and the number of protein–ligand hydrogen bonds. Furthermore, free energy landscape (FEL) maps were constructed using Rg and RMSD as reaction coordinates to visualize the conformational stability of the complexes.

### 2.6 *In vitro* experiments evaluating the inhibitory effects of SD-EVLPs on lung cancer cell proliferation, migration, and invasion

#### 2.6.1 Cell culture

Human lung cancer cell lines A549 and NCI-H1299 were cultured in A549-specific medium (Ham’s F-12K + 10% FBS + 1% P/S) and NCI-H1299-specific medium (RPMI-1640 + 10% FBS + 1% P/S), respectively, in a humidified incubator at 37°C with 5% CO_2_. Cells were passaged every 2–3 days using 0.25% trypsin, and those in the logarithmic growth phase with optimal viability were used for subsequent experiments.

#### 2.6.2 Cellular uptake of SD-EVLPs

A549 and NCI-H1299 cells in the logarithmic growth phase were seeded into 6-well plates at a density of 5 × 10^4^ cells/well. For the experimental group, SD-EVLPs suspensions were mixed with 10 μmol/L DiI fluorescent dye and incubated in the dark for 30 min for membrane labeling. Free dye was removed by centrifugation at 10,000 ×g for 30 min at 4°C, followed by ultracentrifugation at 100,000 ×g for 30 min. The pellet was resuspended in pre-cooled PBS to obtain DiI-labeled SD-EVLPs. For the control group, an equal volume of PBS was processed in parallel instead of SD-EVLPs. The prepared detection solutions were added to the corresponding wells, and cells were co-incubated at 37°C for 24 h. Afterward, cells were washed with pre-cooled PBS and stained with Hoechst working solution for 10 min. Excess dye was removed with PBS, 2 mL of fresh medium was added, and plates were equilibrated for 30 min. All staining procedures were conducted in the dark. Cellular internalization of SD-EVLPs were observed using a fluorescence microscope.

#### 2.6.3 CCK-8 assay

A549 and NCI-H1299 cells in the logarithmic growth phase were seeded into 96-well plates at a density of 5 × 10³ cells per well. Three groups were established: a blank control group containing 100 μL of complete medium, a negative control group containing 100 μL of cell suspension, and treatment groups in which cells were cultured with SD-EVLPs at graded protein concentrations of 0, 3.75, 7.5, 15, 30, 60, 80, or 100 μg/mL. Each group contained six replicate wells, and all plates were incubated under standard culture conditions (37°C, 5% CO_2_) for 24, 48, or 72 h. Thirty minutes before the end of incubation, 10 μL of CCK-8 working solution was added to each well in the dark, and the cells were further incubated until the reaction was complete. Absorbance (*A*) at 450 nm was measured using a multifunctional microplate reader. Cell viability was calculated using GraphPad Prism 9.0 to evaluate cellular activity. Cell viability was calculated as: $\text{Cell viability}(\%)=\frac{A_{\text{SD}-\text{EVLPs}}-A_{\text{blank}}}{A_{\text{Control}}-A_{\text{blank}}}\times 100\%$.

#### 2.6.4 EdU proliferation assay

The EdU incorporation assay was performed to evaluate the effect of SD-EVLPs on cell proliferation. A549 and NCI-H1299 cells in the logarithmic growth phase were seeded into 24-well plates at a density of 3 × 10^4^ cells per well and were treated with SD-EVLPs at various concentrations (0, 15, 30, 60 μg/mL) or cisplatin (5 μmol/L), for 48 h under standard culture conditions. Subsequently, 500 μL of diluted EdU working solution was added to each well, and the cells were incubated for an additional 2 h. After removal of the medium, the cells were fixed with 4% paraformaldehyde for 15 min at room temperature, washed with PBS, permeabilized with 0.3% Triton X-100 for 10 min, and then stained with DAPI for 10 min. Images were captured using a fluorescence microscope, in which EdU-positive cells exhibited green fluorescence and DAPI-stained nuclei appeared blue. The percentage of EdU-positive cells was calculated as: $\text{EdU}-\text{positive rate}(\%)=\frac{\text{Number of EdU}-\text{positive nuclei}}{\text{Total number of nuclei}}\times 100\%$.

#### 2.6.5 Colony formation assay

A colony formation assay was performed to assess the long-term inhibitory effect of SD-EVLPs on cell proliferation. A549 and NCI-H1299 cells in the logarithmic growth phase were seeded into 6-well plates at 500 cells per well and incubated in medium containing SD-EVLPs at various concentrations (0, 15, 30, 60 μg/mL) or cisplatin (5 μmol/L), for ten consecutive days. At the end of treatment, the medium was removed, and the cells were washed three times with PBS, fixed with 4% paraformaldehyde for 15 min at room temperature, and stained with 1% crystal violet for 15 min in the dark. After washing and air-drying, images of whole wells were captured using a digital camera, and colonies containing more than 50 cells were counted using ImageJ software.

#### 2.6.6 Wound healing assay

A wound healing assay was conducted to evaluate the inhibitory effect of SD-EVLPs on cell migration. Three parallel reference lines were drawn on the bottom of each 6-well plate as positional markers. A549 and NCI-H1299 cells were seeded at 5 × 10^5^ cells per well and cultured until they formed a confluent monolayer at approximately 90% confluence. A sterile 200 μL pipette tip was used to create three vertical scratch wounds perpendicular to the reference lines, after which detached cells were washed away with PBS. Fresh medium containing SD-EVLPs at various concentrations (0, 15, 30, 60 μg/mL) or cisplatin (5 μmol/L), was added, and the cells were incubated under standard culture conditions. Images were taken at 0, 24, and 48 h, and wound closure was quantified using ImageJ. The migration rate was calculated as: $\text{Migration rate}(\%)=\frac{\text{Initial wound area}-\text{Final wound area}}{\text{Initial wound area}}\times 100\%$.

#### 2.6.7 Transwell invasion assay

A Transwell invasion assay was used to assess the effect of SD-EVLPs on the invasive capacity of lung cancer cells. Transwell inserts (8 μm pore size) were coated with 60 μL of Matrigel diluted 1:8 in serum-free medium and incubated at 37°C for 3 h. After removing excess Matrigel, the inserts were hydrated with 100 μL serum-free medium for 30 min. A549 and NCI-H1299 cells in the logarithmic growth phase were resuspended in serum-free medium, and 200 μL of cell suspension containing 5 × 10^4^ cells were added to the upper chamber. The lower chamber was filled with 500 μL complete medium containing 10% FBS as a chemoattractant. SD-EVLPs at various concentrations (0, 15, 30, 60 μg/mL) or cisplatin (5 μmol/L), were added to the chambers, and cells were incubated for 48 h under standard conditions. Non-invading cells on the upper surface of the membrane were gently removed with a cotton swab, and the invaded cells on the lower surface were fixed with 4% paraformaldehyde for 30 min and stained with 0.1% crystal violet for 10 min. After washing and drying, cells were imaged under a microscope, and the number of invaded cells was quantified using ImageJ.

### 2.7 *In vitro* experiments verifying the inhibitory effect of SD-EVLPs on lung cancer via the ferroptosis pathway

#### 2.7.1 Detection of Fe²^+^ levels

The intracellular Fe²^+^ levels were assessed using the FerroOrange fluorescent probe. A549 and NCI-H1299 cells in the logarithmic growth phase were seeded in fluorescent culture dishes at a density of 1 × 10^5^ cells/mL and cultured until approximately 60% confluence was reached. Cells were then treated with SD-EVLPs at various concentrations (0, 15, 30, 60 μg/mL) or erastin (20 μmol/L) for 48 h, followed by three washes with serum-free medium. FerroOrange working solution (1 μmol/L, diluted in HBSS solution) was added to the cells. After incubating for 30 min in a 37°C incubator with 5% CO_2_ and 95% humidity, cells were observed and photographed under a fluorescence microscope. Fluorescence intensity was quantified using ImageJ software.

#### 2.7.2 Measurement of MDA and GSH levels

The contents of MDA and GSH in the cells were measured using MDA and GSH detection kits. A549 and NCI-H1299 cells in the logarithmic growth phase were seeded in 6-well plates at a density of 5 × 10^4^ cells/well and cultured in medium containing SD-EVLPs at various concentrations (0, 15, 30, 60 μg/mL) or erastin (20 μmol/L) under standard culture conditions (37°C, 5% CO_2_, 95% humidity) for 48 h. The assays were performed according to the manufacturer’s instructions. *A* values were measured using a microplate reader at wavelengths of 532 nm (for MDA) and 412 nm (for GSH), respectively. The relative concentrations of MDA and GSH in the cells were calculated based on the standard curves.

#### 2.7.3 Detection of intracellular ROS levels

Intracellular ROS levels were measured using the DCFH-DA fluorescent probe. A549 and NCI-H1299 cells in the logarithmic growth phase were seeded into 6-well plates at a density of 5 × 10^4^ cells per well and treated with SD-EVLPs at different protein concentrations (0, 15, 30, 60 μg/mL) or erastin (20 μmol/L) for 48 h under standard culture conditions. The DCFH-DA stock solution was diluted 1:1000 with serum-free medium to prepare a 10 μmol/L working solution, which was added to each well. Cells were incubated for 20 min under standard conditions and subsequently washed three times with serum-free medium to remove excess extracellular probe. Fluorescence images were captured using a fluorescence microscope, and intracellular ROS levels were quantified using ImageJ software.

#### 2.7.4 TEM

TEM was employed to examine mitochondrial ultrastructure. A549 and NCI-H1299 cells treated with SD-EVLPs or erastin were collected, digested with 0.25% trypsin, and fixed in 2.5% glutaraldehyde for 12 h to preserve cellular architecture. Samples were then post-fixed in 1% osmium tetroxide to enhance contrast. A graded series of ethanol–propylene oxide solutions (30–100%) was used for dehydration. Samples were infiltrated with epoxy resin, embedded, and polymerized at 60°C for 48 h. Semi-thin sections were stained with toluidine blue to assess tissue orientation and quality, while ultrathin sections were stained with 2% uranyl acetate followed by lead citrate to enhance contrast. Ultrastructural images were obtained using a transmission electron microscope.

#### 2.7.5 RT-qPCR analysis

RT-qPCR was performed to evaluate the regulatory effect of SD-EVLPs on mRNA expression of key ferroptosis-related genes. A549 and NCI-H1299 cells in the logarithmic growth phase were seeded into 6-well plates at a density of 5 × 10^4^ cells per well and treated with SD-EVLPs at different protein concentrations (0, 15, 30, 60 μg/mL) or erastin (20 μmol/L) for 48 h. Total RNA was extracted using a nucleic acid extraction kit, and RNA concentration and purity were assessed before cDNA synthesis using a reverse transcription kit. qPCR was performed using SYBR Green chemistry, and primer sequences were synthesized by Sangon Biotech (Shanghai) Co., Ltd. (**Table 1**). The amplification program consisted of an initial denaturation at 95°C for 3 min, followed by 40 cycles of denaturation at 95°C for 30 s, annealing at 55°C for 30 s, and extension at 72°C for 30 s. GAPDH served as the internal reference gene. Five biological replicates were included for each group, with three technical replicates per sample. Relative gene expression levels were calculated using the 2⁻^ΔΔCt^ method. mRNA expression of key ferroptosis regulators, including *GPX4* and *SLC7A11*, was quantified, and the expression of *FABP4* and *PPARG* was also examined to further explore candidate molecular changes associated with the SD-EVLP response and ferroptosis-related alterations.

**Table 1:** Primer sequences.

Gene name	Primer sequence (5′~3′)	Product length/bp
*GPX4*	F:CGCTGTGGAAGTGGATGAAGATC R:TGTCGATGAGGAACTGTGGAGAG	112
*SLC7A11*	F:GTCTTCTCCAATTCGGCACCAG R:CCCTCTTCTCCACATACTCCTCTC	102
*FABP4*	F:GGGTGTCCTGGTACATGTGCAG R:CTCTCGTGGAAGTGACGCCTTTC	116
*PPARG*	F:TGAATCCAGAGTCCGCTGACCTC R:ATCGCCCTCGCCTTTGCTTTG	97
*GAPDH*	F:AACATCATCCCTGCCTCTACTGG R:GCCTGCTTCACCACCTTCTTG	182

#### 2.7.6 Western blot analysis

Western blotting was performed to evaluate the expression levels of tumor invasion and metastasis–related proteins (E-cadherin and N-cadherin), key ferroptosis-associated proteins (GPX4 and xCT), and PPAR signaling pathway–related proteins (FABP4 and PPARG) in A549 and NCI-H1299 cells treated with SD-EVLPs at different protein concentrations (0, 15, 30, 60 μg/mL) or erastin (20 μmol/L). After treatment, cells were collected and lysed in RIPA lysis buffer supplemented with 1% PMSF. The lysates were centrifuged, and the supernatants were collected for protein quantification using the BCA assay. Protein samples were mixed with loading buffer, denatured in a 100°C metal bath for 10 min, cooled on ice, and equal amounts of protein were loaded onto sodium dodecyl sulfate–polyacrylamide gel electrophoresis (SDS-PAGE) gels for separation. Following electrophoresis, proteins were transferred onto PVDF membranes using a wet-transfer system with a constant current of 200 mA. The transfer “sandwich” was assembled in the following order: three layers of filter paper, the PVDF membrane, the gel, and another three layers of filter paper. After transfer, membranes were blocked with 5% non-fat milk at room temperature for 1 h and subsequently incubated overnight at 4°C with the following primary antibodies: E-cadherin (1:10,000), N-cadherin (1:1,000), xCT (1:4,000), GPX4 (1:4,000), FABP4 (1:3,000), PPARG (1:2,000) and β-actin (1:5,000). Membranes were washed three times with TBST (10 min each), incubated with HRP-conjugated secondary antibodies (1:5,000) at room temperature for 1 h, and washed again three times with TBST (15 min each). Protein bands were visualized using an enhanced chemiluminescence (ECL) substrate, and images were captured with a gel imaging system. β-actin was used as the internal loading control, and ImageJ software was employed for quantitative analysis of the relative expression levels of each target protein.

### 2.8 *In vivo* experiments to evaluate the effect of SD-EVLPs on subcutaneous lung cancer xenografts

#### 2.8.1 Animal grouping, tumor modeling, and drug administration

BALB/c nude mice were acclimated for 7 days. A549 cells were resuspended in serum-free medium at 5 × 10^7^ cells/mL, and 1 × 10^7^ cells were subcutaneously injected into the right axillary region of each mouse. Seven days after inoculation, 25 mice were randomized into five groups (n = 5 per group): control (normal saline), SD-EVLP low-dose (SD-EVLPs-L, 2 mg/kg), SD-EVLP high-dose (SD-EVLPs-H, 4 mg/kg), cisplatin positive control (5 mg/kg), and the ferroptosis positive control IKE (30 mg/kg) (17). SD-EVLPs were administered intraperitoneally every other day for 14 days; cisplatin was administered once per week for 14 days; and IKE was administered intraperitoneally every 3 days for 14 days. The injection volume of SD-EVLPs was calculated based on the formulation protein concentration and adjusted according to individual body weight. A preliminary toxicity study (n = 3 per group) was conducted prior to efficacy experiments to confirm tolerability of the dosing regimen (18). Cisplatin was used as a clinically relevant chemotherapy comparator to benchmark *in vivo* antitumor efficacy, whereas IKE, an optimized erastin derivative with improved *in vivo* suitability, served as a ferroptosis-specific inducer control to probe the mechanism specificity of SD-EVLP–associated ferroptosis in tumors. Notably, IKE was included only for validating ferroptosis-related biomarkers in tumor tissues and was not included in the primary antitumor efficacy comparisons.

#### 2.8.2 Nude mice body weight and tumor volume

During the treatment period, nude mice in each group had free access to food and water, and their body weights were measured every three days. After 14 days of intervention, the mice were euthanized, and subcutaneous tumors were excised, photographed, and measured for length and width. Tumor volume was calculated using the formula: $\text{Tumor volume=}\frac{{\text{length×width}}^{2}}{2}$. In addition, heart, liver, lung, and kidney tissues were collected from each mouse for subsequent analyses.

#### 2.8.3 Evaluation of liver and kidney function

Blood samples were collected from nude mice, and serum levels of alanine aminotransferase (ALT), aspartate aminotransferase (AST), blood urea nitrogen (BUN), and creatinine (CREA) were measured to assess the effects of SD-EVLPs and cisplatin on hepatic and renal function.

#### 2.8.4 Hematoxylin–eosin staining

H&E staining was performed to evaluate pathological alterations in tumor tissues. Tumor samples were fixed in 4% paraformaldehyde and subsequently processed through routine histological procedures, including dehydration, clearing, paraffin embedding, sectioning, and dewaxing. Paraffin sections of approximately 4μm thickness were immersed in hematoxylin solution for 3 min, rinsed, and then stained with eosin for 5 min. After a second rinse, the sections were dehydrated, cleared, mounted with neutral resin, and examined under a light microscope. Histopathological assessment included evaluation of cellular atypia, mitotic figures, necrosis, stromal infiltration, and angiogenesis. To further assess the systemic biosafety of SD-EVLPs and cisplatin, heart, liver, lung, and kidney tissues were processed using the same H&E staining protocol. Microscopic examination focused on hepatocyte cord structure, myocardial fiber arrangement, alveolar integrity, and renal tubular–glomerular morphology, with particular attention to any evidence of necrosis, hemorrhage, steatosis, or inflammatory infiltration.

#### 2.8.5 Immunohistochemistry

IHC was performed to assess Ki-67 expression in tumor tissues as an indicator of proliferative activity. Paraffin-embedded tumor sections were baked, dewaxed in xylene, rehydrated through a graded ethanol series, and rinsed with PBS. Antigen retrieval was carried out by incubating the sections in citrate buffer. Subsequently, the slides were incubated with 3% hydrogen peroxide in the dark to quench endogenous peroxidase activity, followed by three washes with PBS on a shaker to remove excess hydrogen peroxide.

After blocking with serum for 30 min, the sections were incubated with an anti-Ki-67 primary antibody (1:200) at 4°C overnight. On the following day, the slides were incubated with an HRP-conjugated goat anti-mouse/rabbit secondary antibody at room temperature for 50 min. DAB was used for chromogenic development, followed by hematoxylin counterstaining. The sections were then dehydrated, cleared, mounted with neutral resin, and examined and photographed under a light microscope. Quantitative analysis was conducted using ImageJ software. The Ki-67 positivity rate was calculated as: $\text{Positive cell ratio }(\%)=\frac{\text{Number of positive nuclei}}{\text{Total number of nuclei}}\times 100\%$.

#### 2.8.6 Detection of Fe²^+^, GSH, and MDA levels

The levels of Fe²^+^, GSH, and MDA in tumor tissues were measured using a ferrous ion colorimetric assay kit, a GSH detection kit, and an MDA detection kit, respectively, following the manufacturers’ instructions.

#### 2.8.7 Western blot analysis

Western blot analysis was performed to evaluate the protein expression levels of key ferroptosis-related regulators (GPX4 and xCT) and PPAR-associated pathway proteins (FABP4 and PPARG) in tumor tissues from nude mice treated with different doses of SD-EVLPs or IKE. Experimental procedures followed the protocol described in Section 2.7.6.

### 2.9 Statistical analysis

Statistical analyses were performed using the “stats” and “car” packages in R software (version 4.3.0), and data visualization was conducted using the “ggplot2” package. All results are presented as mean ± standard error (SEM). Continuous variables were tested for normality using the Shapiro–Wilk test and for homogeneity of variance using Levene’s test. For data meeting both assumptions, one-way ANOVA followed by Tukey’s HSD *post hoc* test was performed. For normally distributed data with unequal variances, Welch’s one-way ANOVA followed by the Games–Howell *post hoc* test was used. For non-normally distributed data, the Kruskal–Wallis test followed by Dunn’s test was applied. A *P*-value < 0.05 was considered statistically significant.

## 3 Results

### 3.1 Characterization and active component screening of SD-EVLPs

#### 3.1.1 Characterization of SD-EVLPs

**Figure 1:**
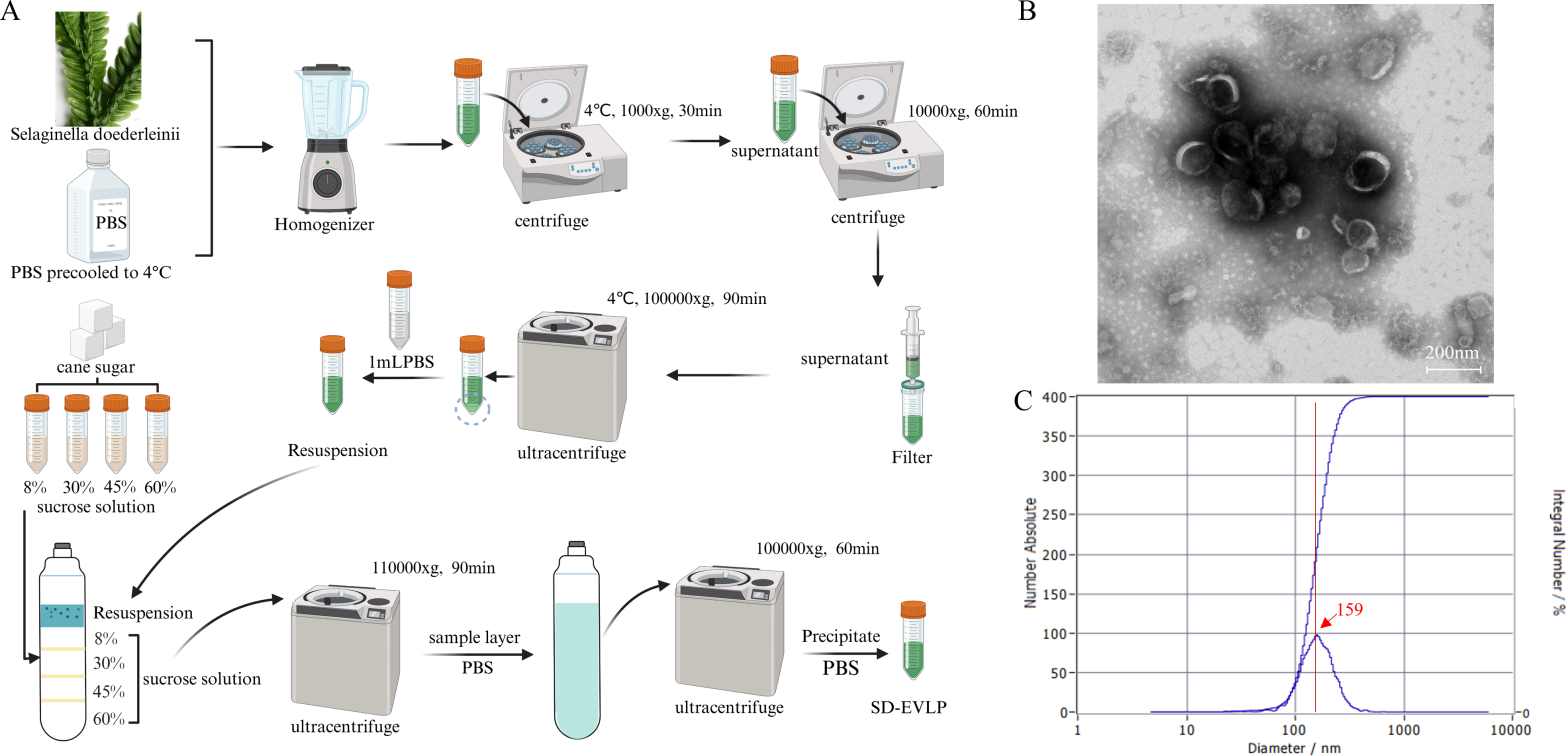
Extraction, purification, and characterization of SD-EVLPs **(A)** Extraction and purification procedure of SD-EVLPs; **(B)** TEM observation of SD-EVLPs morphology; **(C)** NTA detection of SD-EVLPs particle size distribution.

SD-EVLPs were successfully extracted and purified using a combined differential centrifugation and ultracentrifugation approach followed by sucrose gradient centrifugation. TEM revealed spherical or oval vesicle-like particles with uniform and intact morphology. NTA showed that SD-EVLPs had a mean hydrodynamic diameter of 159 nm with a highly concentrated size distribution. The BCA assay further demonstrated that the protein concentration of the purified SD-EVLPs was 401.03 μg/mL (**Figure 1**).

#### 3.1.2 Active components of SD-EVLPs

LC-MS analysis revealed that SD-EVLPs contained 93 compounds, including Shogaol, Tropine, and Amentoflavone. The Base Peak Chromatograms (BPC) were obtained in both positive and negative ion modes, with 49 compounds identified in the positive ion mode and 44 compounds detected in the negative ion mode. Component classification showed that SD-EVLPs includes: 1 amino acid and peptide, 4 phenylpropanoids, 6 phenols, 7 flavonoids, 1 quinone, 3 alkaloids, 2 carboxylic acids and derivatives, 15 sugars and glycosides, 11 terpenoids, 1 indole and its derivative, 4 organic acids and derivatives, 7 organic heterocyclic compounds, 4 steroids, 18 fatty acyls, and 9 other compounds, confirming that SD-EVLPs contains multiple bioactive components.

**Figure 2:**
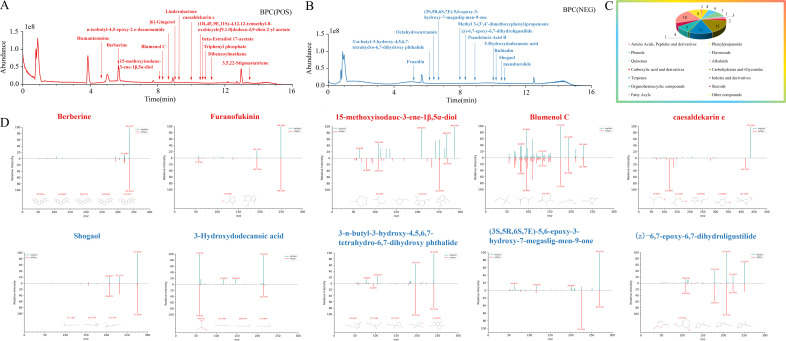
Active components of SD-EVLPs **(A)** BPC of SD-EVLPs in positive ion mode; **(B)** BPC of SD-EVLPs in negative ion mode; **(C)** Quantity distribution map of component classification of SD-EVLPs; **(D)** MS/MS spectra of 10 active components in SD-EVLPs.

Based on the stringent screening criteria from the TCMSP and SwissADME platforms, 25 active components, including Shogaol, Berberine, and 3-Hydroxydodecanoic acid, were identified from the 93 compounds. These include 2 phenylpropanoids, 3 phenols, 1 flavonoid, 1 quinone, 1 alkaloid, 2 carboxylic acids and derivatives, 5 terpenoids, 1 indole and its derivative, 1 organic acid and derivative, 3 organic heterocyclic compounds, and 2 steroids. The tandem mass spectrometry (MS/MS) spectra of 10 representative active components were displayed (**Figure 2**).

### 3.2 Bioinformatics analysis results

#### 3.2.1 Analysis of SD-EVLPs intervention targets and their expression in lung cancer patients

Venn diagram–based intersection analysis identified 17 ferroptosis-related targets of SD-EVLPs intervention in lung cancer, including FABP4, IL6, ALOX5, PPARG, TLR4, AURKA, MMP13, CDC25A, ADORA2B, NOS2, ENPP2, AR, ERN1, PDGFRA, JUN, CD38, and TRPV1. Paired and unpaired t-test validation using TCGA-LUAD and TCGA-LUSC cohorts demonstrated that all 17 genes exhibited significant differential expression between lung cancer tissues and adjacent normal tissues (*P* < 0.001). Among these genes, 12, such as FABP4, IL6, and ALOX5, were significantly downregulated in lung cancer tissues, whereas 5 genes, including AURKA, MMP13, and CDC25A, were significantly upregulated, suggesting their potential tumor-suppressive or oncogenic functions, respectively.

To identify prognosis-related SD-EVLPs intervention targets, LASSO regression combined with ten-fold cross-validation was performed on the 17 genes, resulting in the selection of 8 key genes with strong prognostic relevance. These genes were FABP4, IL6, PPARG, AURKA, NOS2, PDGFRA, JUN, and TRPV1. The likelihood deviance values corresponding to different lambda parameters were visualized to illustrate the coefficient shrinkage process and the relative contribution of each gene to prognosis.

**Figure 3:**
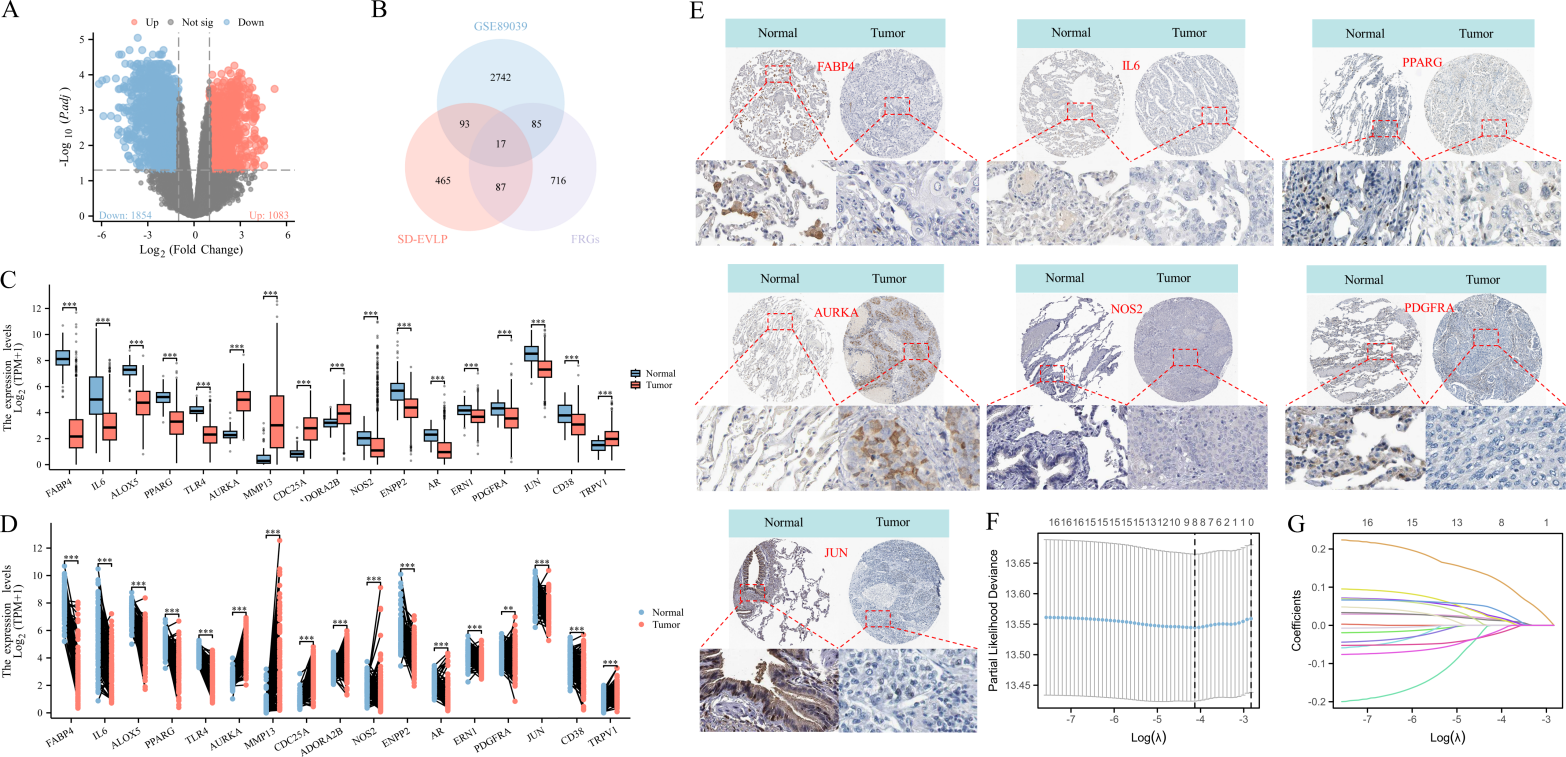
Acquisition and differential expression analysis of SD-EVLPs intervention targets **(A)** Volcano plot of differential analysis for GSE89039; **(B)** Venn diagram showing the intersection of SD-EVLPs intervention targets, differential genes from GSE89039 lung cancer data, and ferroptosis-related genes; **(C)** Unpaired expression analysis of SD-EVLPs intervention targets in lung cancer patients; **(D)** Paired expression analysis of SD-EVLPs intervention targets in lung cancer patients; **(E)** Immunohistochemical analysis of SD-EVLPs intervention targets in the HPA database; **(F, G)** LASSO regression analysis of SD-EVLPs intervention targets.

Furthermore, protein expression levels of the selected targets were validated using the HPA database. The results showed that FABP4, IL6, PPARG, NOS2, PDGFRA, and JUN were markedly downregulated in lung cancer tissues, while AURKA was markedly upregulated, which was highly consistent with expression trends observed in TCGA transcriptomic data. It is noteworthy that immunohistochemistry data for TRPV1 are not yet available in the HPA database; therefore, TRPV1 could not be included in this round of protein-level validation (**Figure 3**).

#### 3.2.2 Prognostic analysis of SD-EVLPs intervention targets in lung cancer patients

Based on survival data from the TCGA-LUAD and TCGA-LUSC cohorts, with OS as the time variable and survival status as the endpoint, univariate and multivariate Cox proportional hazards regression analyses were conducted on the prognostic-related SD-EVLP intervention targets. According to the median risk score, 1,026 lung cancer patients were stratified into a high-risk group (n = 513) and a low-risk group (n = 513). Survival analysis revealed a significant difference in prognosis between the two groups (*P* = 0.01). Patients in the high-risk group had poorer overall survival compared with those in the low-risk group (HR = 1.30, 95% CI [1.06, 1.58]). These findings suggest that the multi-gene scoring system may provide supportive prognostic stratification information for patients with lung cancer.

**Figure 4:**
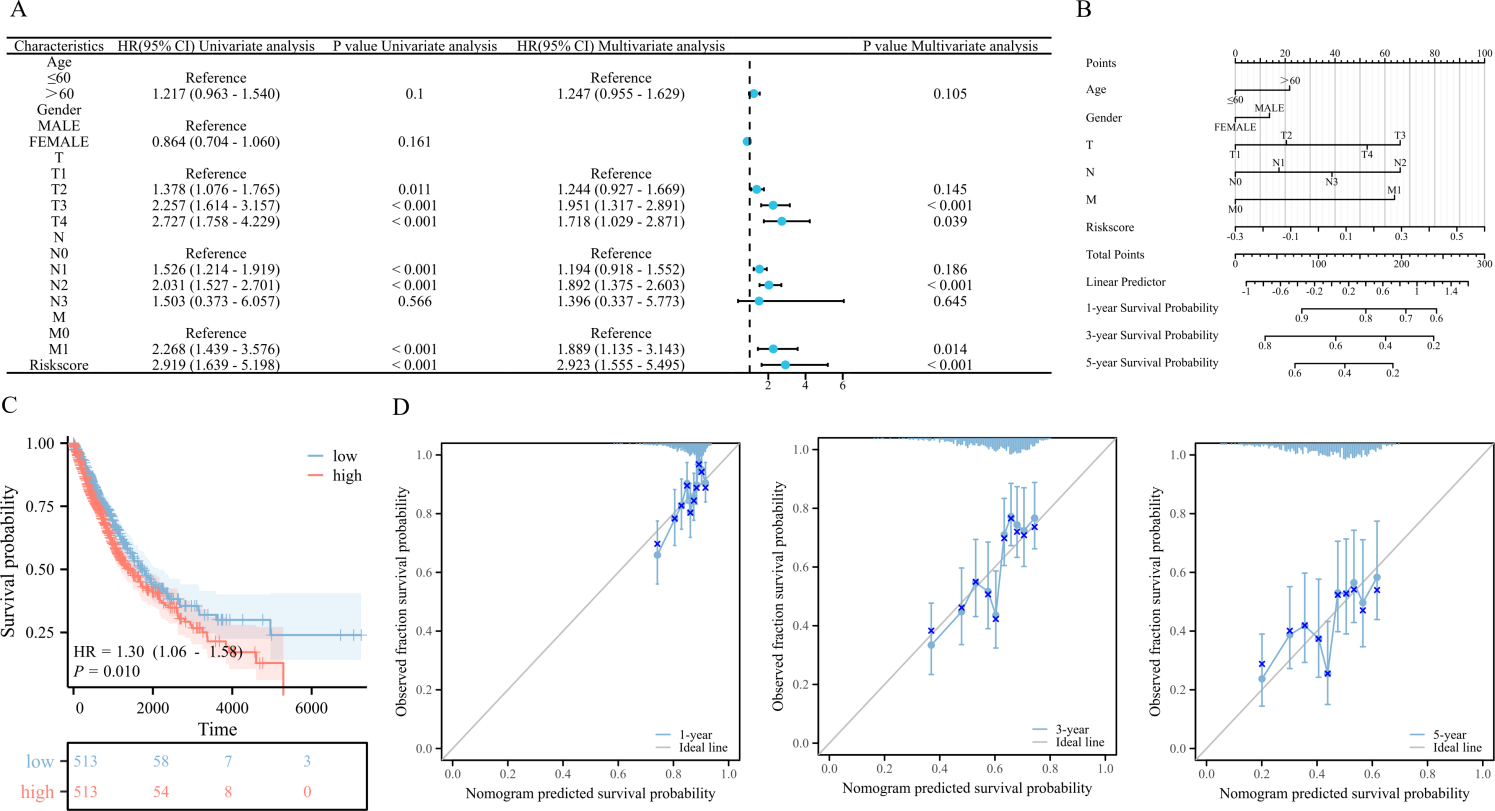
Supportive prognostic analysis of prioritized SD-EVLP intervention targets in lung cancer **(A)** Forest plot of univariate and multivariate Cox regression for SD-EVLPs intervention targets; **(B)** Nomogram constructed using clinical information and risk score; **(C)** Kaplan–Meier survival curve of the risk scoring system; **(D)** Calibration curves for 1-, 3-, and 5-year survival predictions of the Nomogram.

Clinical variables and risk scores of lung cancer patients were further incorporated into univariate and multivariate Cox regression analyses. Univariate Cox regression identified age, TNM stage, and risk score as variables significantly associated with survival outcomes (*P* < 0.1). Further multivariate Cox analysis confirmed that T3 stage (HR = 1.951, 95% CI [1.317, 2.891], *P* < 0.001), N2 stage (HR = 1.892, 95% CI [1.375, 2.603], *P* = 0.006), and the risk score (HR = 2.92, 95% CI [1.56, 5.50], *P* < 0.001) were independent prognostic risk factors. A forest plot was generated to visually demonstrate the independent predictive effects of these variables. Based on the identified prognostic factors, a nomogram incorporating age, TNM stage, and risk score was constructed to predict 1-, 3-, and 5-year survival probabilities in lung cancer patients. The concordance index (C-index) of the model reached 0.64 (95% CI [0.624, 0.658]), indicating favorable predictive performance and providing supportive clinical relevance for the prioritized candidate targets (**Figure 4**).

#### 3.2.3 Network pharmacology, molecular docking, and MD simulation of SD-EVLPs intervention targets

Based on the PPI network constructed from the STRING database, 17 SD-EVLPs target proteins formed 91 PPI pairs, with the highest degree values found for IL6, PPARG, and JUN, which constitute the core hubs of the network. GO and KEGG enrichment analyses were performed with a threshold of *P* < 0.05, and the results were visualized in bubble plots. The GO analysis indicated that the main biological processes affected by SD-EVLPs intervention include response to oxidative stress, regulation of protein secretion, cytokine production in inflammatory responses, hormone secretion regulation, and macrophage activation. Molecular functions involved platelet-derived growth factor receptor binding, fatty acid binding, nuclear receptor activity, and calcium release channel activity. The KEGG enrichment analysis revealed that the major pathways through which SD-EVLPs exert its anti-lung cancer effects include chemical carcinogenesis-receptor activation, the PPAR signaling pathway, EGFR tyrosine kinase inhibitor resistance, and the expression of PD-L1 and the PD-1 checkpoint pathway in cancer, suggesting that SD-EVLPs holds therapeutic potential in treating lung cancer.

**Figure 5:**
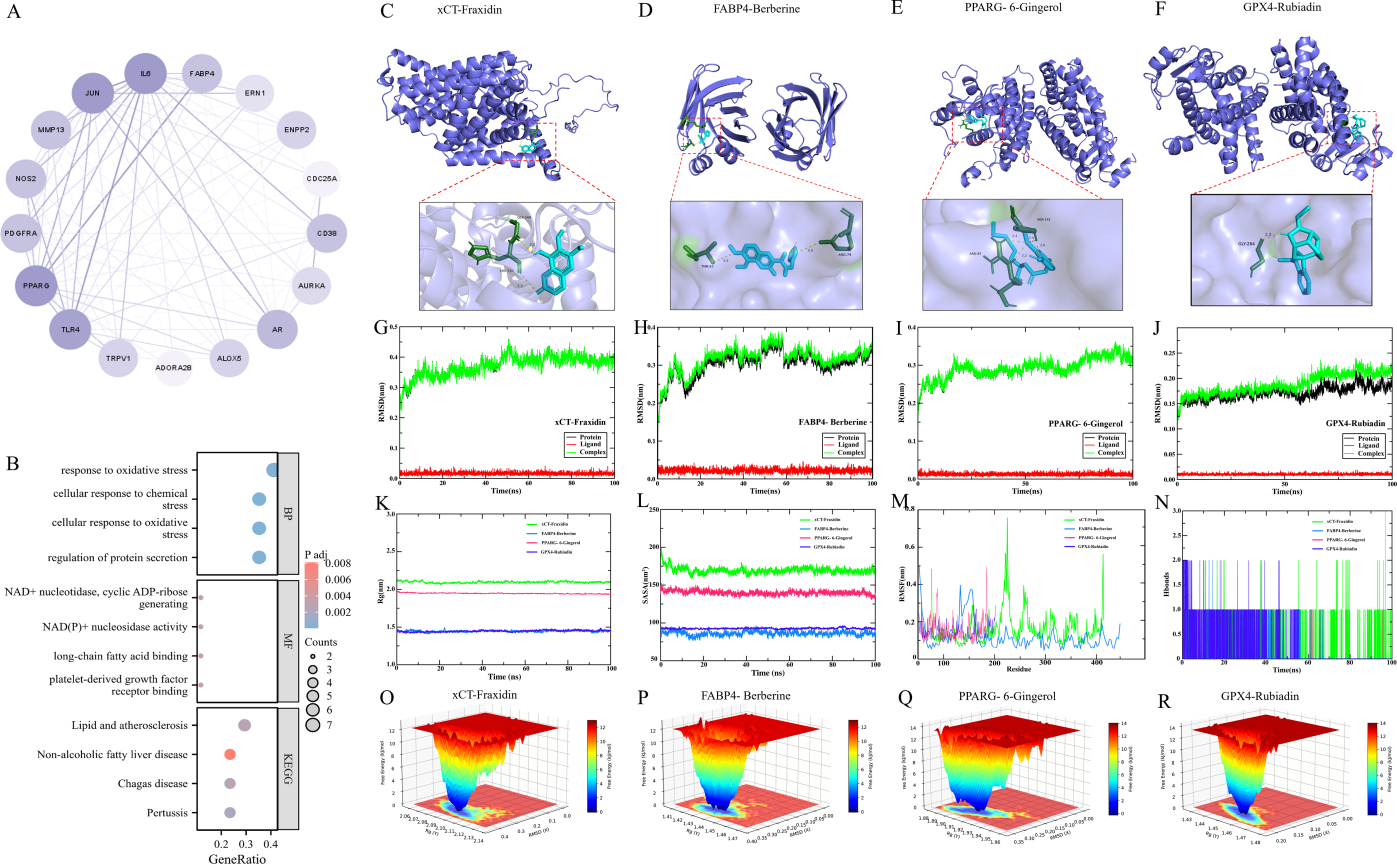
Network pharmacology, molecular docking, and MD simulation of SD-EVLPs intervention targets **(A)** Construction of the PPI network for the 17 SD-EVLPs intervention targets; **(B)** GO and KEGG enrichment analysis results; **(C–F)** Molecular docking results of SD-EVLPs active components with pathway-related proteins; **(G–N)** All-atom 100 ns MD simulations evaluating complex stability and flexibility, including RMSD **(G–J)**, Rg **(K)**, SASA **(L)**, residue RMSF **(M)**, and intermolecular hydrogen bond counts **(N)**; O–R: FEL.

Based on the KEGG enrichment results, we prioritized the FABP4/PPARG/GPX4 regulatory axis that was enriched in the PPAR signaling pathway. This pathway is closely linked to lipid metabolic homeostasis and ferroptosis vulnerability, and database validation further indicated that FABP4, PPARG, and GPX4 were significantly associated with lung cancer prognosis, supporting their translational relevance. Nevertheless, other candidate pathways may also contribute to the bioactivity of SD-EVLPs and warrant systematic investigation in future studies. Focusing on the PPAR signaling pathway, particularly the FABP4/PPARG/GPX4 regulatory axis, which was enriched in KEGG analysis, we employed molecular docking to assess the interaction mechanisms between the active components of SD-EVLPs and key targets. The results revealed strong binding affinities between the effective components of SD-EVLPs and the proteins FABP4, PPARG, GPX4, and xCT. Specifically, Berberine, an active component of SD-EVLPs, showed a binding energy of -8.7 kcal/mol with FABP4, forming hydrogen bonds with THR-57 (3.5 Å) and ARG-79 (3.3Å). Similarly, 6-Gingerol exhibited a binding energy of -7.6 kcal/mol with PPARG, forming hydrogen bonds with SER-141 (2.4 Å, 2.0 Å) and ARG-87 (2.4 Å, 2.2 Å). Rubiadin, another component, bound to GPX4 with a binding energy of -8.1 kcal/mol, interacting with GLY-284 (2.2 Å) through hydrogen bonds. Additionally, Fraxidin, an effective component, demonstrated a binding energy of -8.2 kcal/mol with xCT, forming hydrogen bonds with HIS-350 (3.3 Å) and GLY-349 (2.5 Å). These results suggest that SD-EVLPs may exert anti-lung cancer effects in association with ferroptosis-related changes and modulation of the FABP4/PPARG/GPX4-associated pathway. We present the docking results of the four active components of SD-EVLPs with the pathway proteins. Molecular docking is a widely used computational method to study the interaction between small molecules and target proteins, predicting the binding conformation and affinity of ligands at receptor active sites. However, traditional docking methods often use rigid or semi-rigid models, which fail to fully account for the flexibility of molecular conformations under physiological conditions and dynamic changes in the solution environment. To more comprehensively evaluate the binding stability and interaction characteristics of small molecule-protein complexes, we conducted full-atom MD simulations based on molecular docking to model the conformational evolution of complexes under near-physiological conditions. By monitoring key dynamic parameters such as RMSD, RMSF, Rg, SASA, and hydrogen bond numbers, we systematically analyzed the structural stability, flexibility, and interaction strength of the complexes, providing theoretical support for understanding the binding mechanisms between small molecules and target proteins. For the four complexes: xCT-Fraxidin, FABP4-Berberine, PPARG-6-Gingerol, and GPX4-Rubiadin, the RMSD of the complexes generally remained below 0.5nm, and the RMSD of the ligands in each system was less than 0.05nm, indicating strong pocket constraints. This suggests that each small molecule binds with its target protein with high stability. The radius of Rg of all complexes was in the range of 1.25-2.25nm, with minimal fluctuation over time. SASA analysis revealed that xCT-Fraxidin exhibited a larger surface exposure, whereas FABP4-Berberine had the smallest, indicating that the latter may have a deeper pocket or a denser complex. The RMSF of residues was mostly below 0.5nm, with xCT-Fraxidin showing peaks in the loop or terminal regions, reflecting local flexibility. Regarding hydrogen bond occupancy, the number of hydrogen bonds between small molecules and target proteins ranged from 0 to 2, with approximately one hydrogen bond in most cases. This suggests good hydrogen bonding interactions between the ligands and their target proteins. To further explore the dynamic conformational stability and intrinsic interaction mechanisms of the protein-complex systems, we constructed 3D FEL to analyze the energy distribution characteristics in the conformational space. Using Rg and RMSD as reaction coordinates, the free energy changes during the conformational evolution were quantitatively represented. Analysis showed that the four complexes, xCT-Fraxidin, FABP4-Berberine, PPARG-6-Gingerol, and GPX4-Rubiadin, each displayed concentrated energy minima, indicating that they possess stable, aggregated low-energy conformations and stable binding modes (**Figure 5**).

### 3.3 SD-EVLPs inhibit proliferation, migration, and invasion of lung cancer cells

#### 3.3.1 Internalization of SD-EVLPs by lung cancer cells

**Figure 6:**
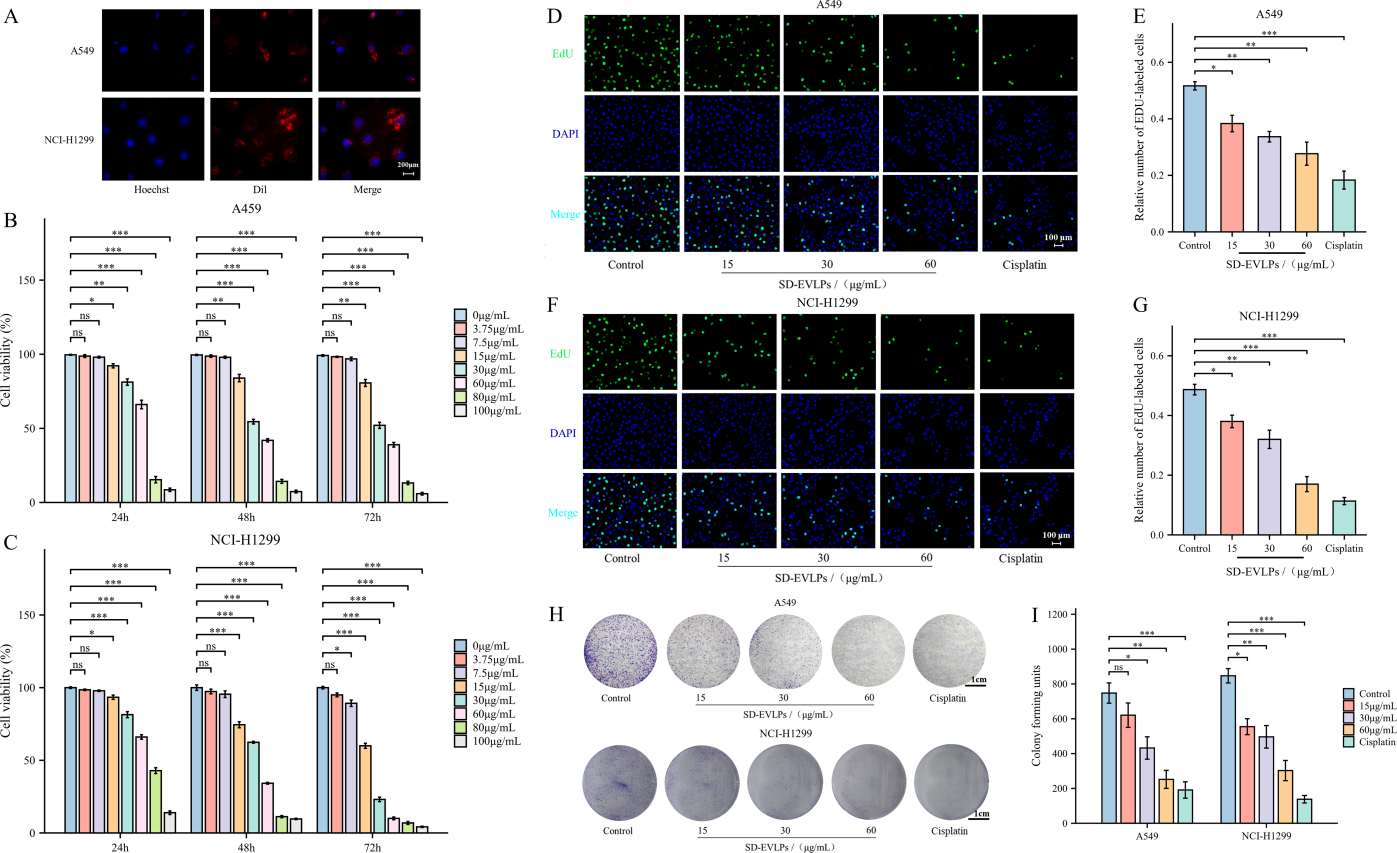
Internalization of SD-EVLPs and its inhibitory effects on lung cancer cell proliferation **(A)** Internalization of SD-EVLPs by lung cancer cells; **(B, C)** CCK-8 assays assessing the effects of SD-EVLPs on lung cancer cell viability; **(D–G)** EdU assays evaluating the proliferation of A549 and NCI-H1299 cells; **(H–I)** Colony formation assays assessing the proliferative capacity of A549 and NCI-H1299 cells. Data are presented as the mean ± SEM, n = 3 independent experiments. Compared with the control group, *P < 0.05, **P < 0.01, and ***P < 0.001.

PDEVs contain various essential biomolecules, including proteins, nucleic acids, and lipids (19). They are internalized by recipient cells primarily through endocytosis and play important roles in intercellular communication and cellular regulatory processes (20, 21). Therefore, evaluating the uptake of PDEVs by tumor cells is of particular importance. DiI, a lipophilic fluorescent dye, specifically labels vesicles with lipid bilayer membranes. In the cellular uptake assay, strong granular red fluorescence signals were observed in A549 and NCI-H1299 cells following SD-EVLP treatment, whereas no comparable fluorescence was detected in the control group. These findings demonstrate that SD-EVLPs can be effectively internalized by both A549 and NCI-H1299 lung cancer cells (**Figure 6A**).

#### 3.3.2 SD-EVLPs inhibit the proliferation of lung cancer cells

The CCK-8 assay was used to evaluate the effect of SD-EVLPs on the viability of A549 and NCI-H1299 cells. The results showed that treatment with low protein concentrations of SD-EVLPs for 24, 48, or 72 h did not cause significant changes in the viability of either cell line. However, when the SD-EVLPs protein concentration reached 15 μg/mL, cell viability was significantly reduced at all examined time points (*P* < 0.05, *P* < 0.01, *P* < 0.001). Moreover, increasing concentrations of SD-EVLPs led to a dose-dependent decrease in cell viability in both A549 and NCI-H1299 cells, indicating a concentration-dependent inhibitory effect on lung cancer cell proliferation (*P* < 0.05, *P* < 0.01, *P* < 0.001).

To systematically assess the inhibitory effect of SD-EVLPs on lung cancer cell proliferation, EdU incorporation assays and colony formation assays were performed on A549 and NCI-H1299 cells. The EdU assay results demonstrated that exposure to 15, 30, or 60 μg/mL SD-EVLPs or cisplatin for 48 h significantly suppressed cellular proliferation in a concentration-dependent manner. Notably, treatment with medium and high protein concentrations of SD-EVLPs (30 and 60 μg/mL) markedly reduced the proportion of EdU-positive cells, and the differences compared with the control group were highly significant (*P* < 0.01, *P* < 0.001). In the colony formation assay, treatment with SD-EVLPs at 30 or 60 μg/mL for 10 days significantly inhibited colony-forming ability in both A549 and NCI-H1299 cell lines, as evidenced by a substantial reduction in colony numbers (*P* < 0.05, *P* < 0.01, *P* < 0.001). Together, these findings clearly demonstrate that SD-EVLPs exert a pronounced, concentration-dependent inhibitory effect on the proliferation of lung cancer cells (**Figures 6B–I**).

#### 3.3.3 SD-EVLPs inhibit the migration and invasion of lung cancer cells

Wound healing and Transwell assays were conducted to assess the effects of SD-EVLPs on the migration and invasion of A549 and NCI-H1299 cells. The wound healing assay demonstrated that, compared with the control group, treatment with SD-EVLPs at 15, 30, or 60 μg/mL for 24 h and 48 h markedly reduced the number of cells migrating into the scratched region. The migration rate decreased progressively as the SD-EVLPs concentration increased. Consistently, the number of cells penetrating the Transwell membrane was significantly reduced in a concentration-dependent manner. Notably, medium and high concentrations of SD-EVLPs (30, 60 μg/mL) exerted significant inhibitory effects on both migration and invasion (*P* < 0.05, *P* < 0.01, *P* < 0.001). These findings indicate that SD-EVLPs effectively suppress the migratory and invasive capacities of A549 and NCI-H1299 lung cancer cells.

**Figure 7:**
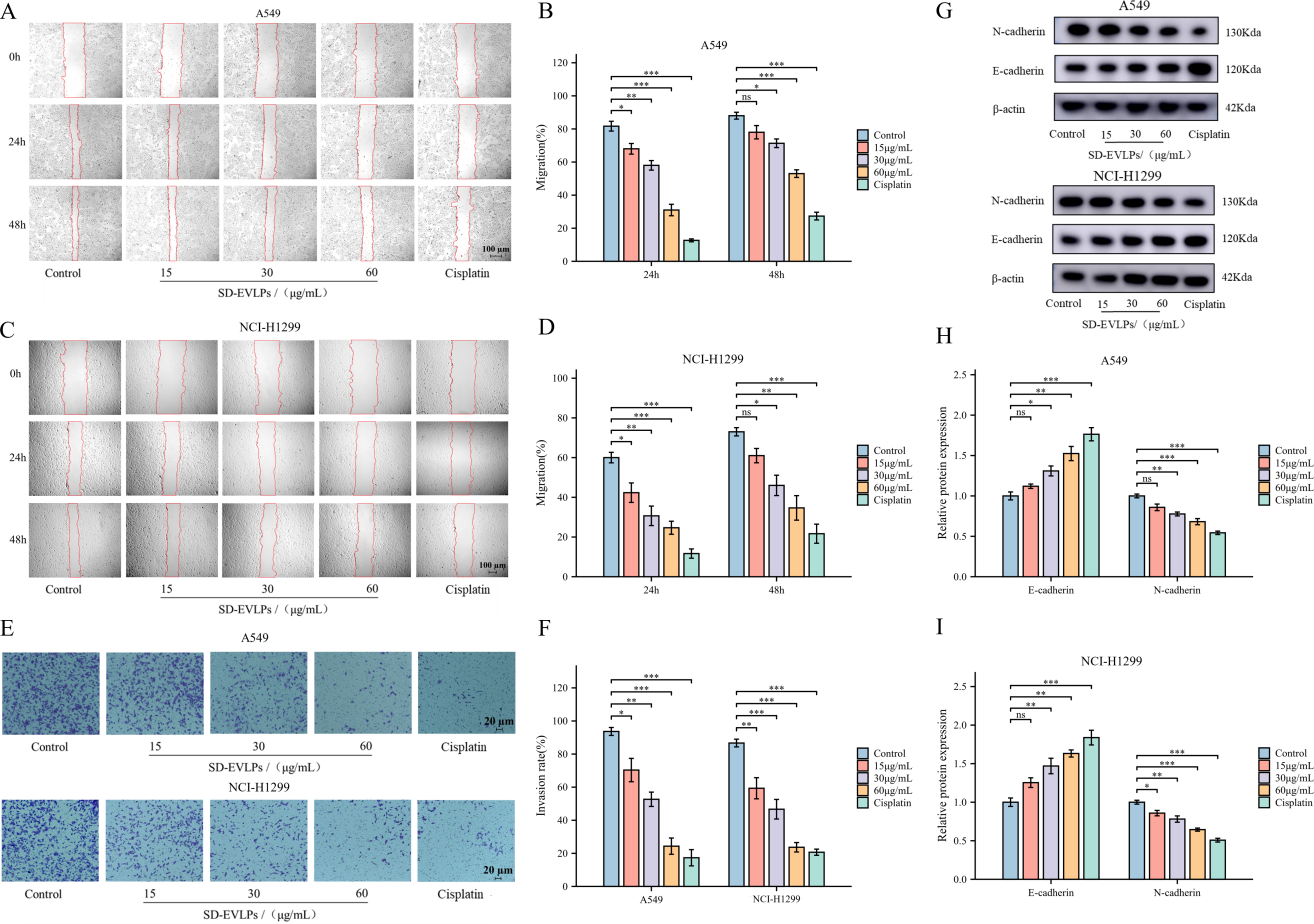
SD-EVLPs inhibit migration and invasion of lung cancer cells **(A–D)** Wound healing assays assessing the effect of SD-EVLPs on the migration of A549 and NCI-H1299 cells; **(E, F)** Transwell assays evaluating the invasion ability of A549 and NCI-H1299 cells; **(G–I)** Western blot analysis of E-cadherin and N-cadherin protein expression. Data are presented as the mean ± SEM, n = 3 independent experiments. Compared with the control group, *P < 0.05, **P < 0.01, and ***P < 0.001.

E-cadherin loss and N-cadherin gain are hallmark features of epithelial–mesenchymal transition (EMT), which is closely associated with enhanced migratory and invasive behavior in cancer cells. Western blot analysis revealed that SD-EVLPs dose-dependently increased the epithelial marker E-cadherin and decreased the mesenchymal marker N-cadherin in both A549 and NCI-H1299 cells compared with the control group. These EMT-related changes are consistent with the functional inhibition of migration and invasion, supporting that SD-EVLPs suppress metastasis-associated phenotypes in lung cancer cells (**Figure 7**).

### 3.4 *In vitro* validation of SD-EVLPs-promoted ferroptosis in lung cancer cells

#### 3.4.1 SD-EVLPs promote ferroptosis in lung cancer cells

**Figure 8:**
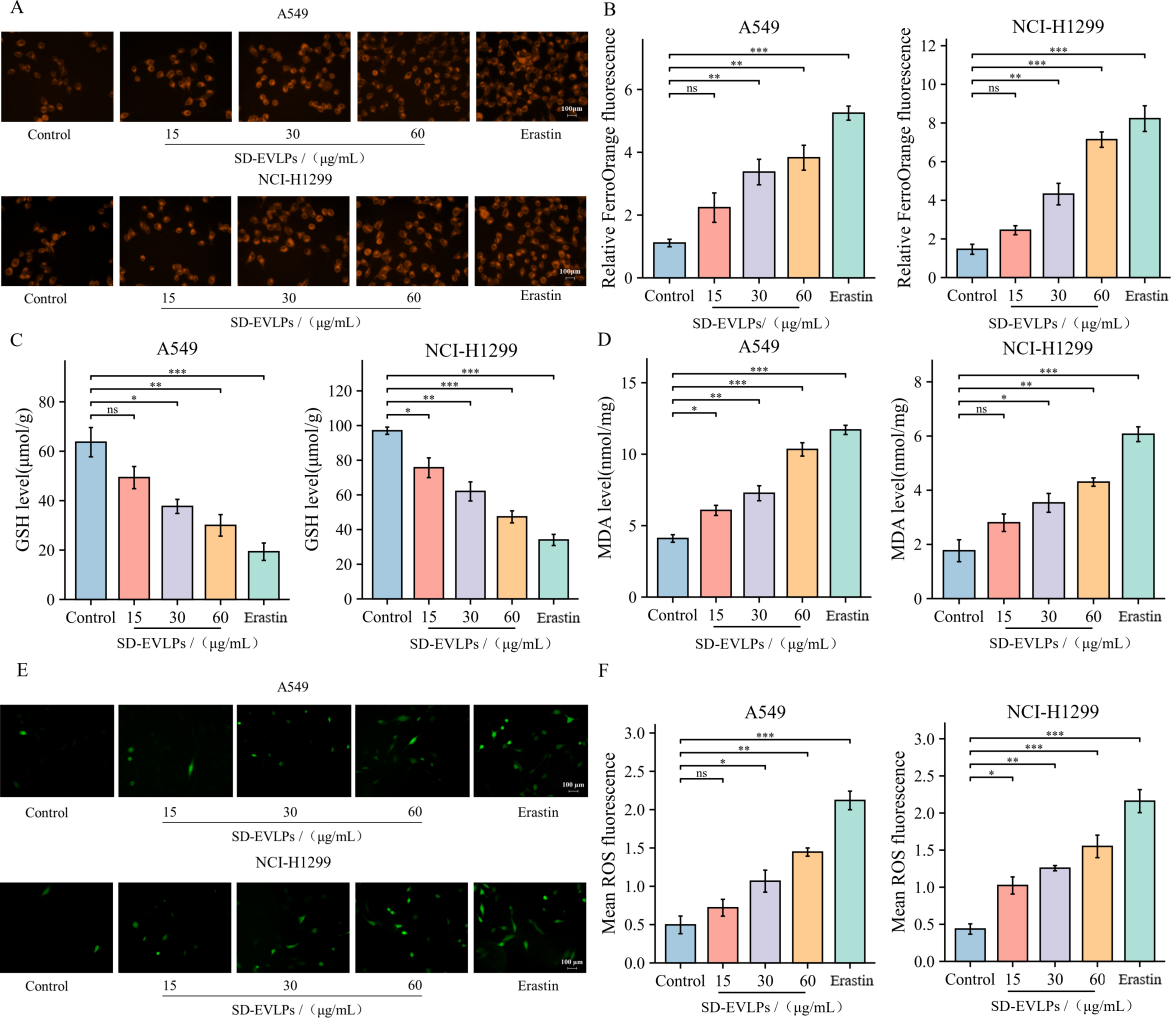
Measurement of ferroptosis-related indicators (Fe²^+^, GSH, MDA, ROS) in lung cancer cells treated with SD-EVLPs **(A, B)** Intracellular Fe²^+^ levels; **(C)** GSH levels; **(D)** MDA levels; **(E, F)** Intracellular ROS levels. Data are presented as the mean ± SEM, n = 3 independent experiments. Compared with the control group, *P < 0.05, **P < 0.01, and ***P < 0.001.

Ferroptosis, an iron-dependent form of programmed cell death, is characterized by excessive accumulation of lipid peroxidation products. In this study, a comprehensive *in vitro* assessment system was employed to evaluate the regulatory effects of SD-EVLPs on ferroptosis-related biomarkers in lung cancer cells. Treatment with medium and high protein concentrations of SD-EVLPs (30 and 60 μg/mL), as well as erastin (20 μmol/L), significantly increased intracellular Fe²^+^, MDA, and ROS levels while markedly reducing GSH levels in both A549 and NCI-H1299 cells compared with the control group (*P* < 0.01, *P* < 0.001). These results suggest that SD-EVLPs at these concentrations may promote ferroptosis by elevating intracellular Fe²^+^ and ROS levels, inhibiting peroxidase activity, and promoting lipid peroxidation in lung cancer cells (**Figure 8**).

At the molecular level, RT-qPCR results showed that, compared with the control group, treatment with medium and high concentrations of SD-EVLPs (30 and 60 μg/mL) or erastin (20 μmol/L) significantly suppressed the mRNA expression levels of *GPX4* and *SLC7A11* in A549 and NCI-H1299 cells (*P* < 0.05, *P* < 0.01, *P* < 0.001). Western blot analysis further confirmed that SD-EVLPs decreased the protein expression of GPX4 and xCT in a dose-dependent manner. Medium and high concentrations of SD-EVLPs (30 and 60 μg/mL), as well as erastin, markedly reduced GPX4 and xCT protein expression in both lung cancer cell lines (*P* < 0.05, *P* < 0.01, *P* < 0.001), indicating that SD-EVLPs treatment is associated with coordinated alterations in key ferroptosis-related proteins.

**Figure 9:**
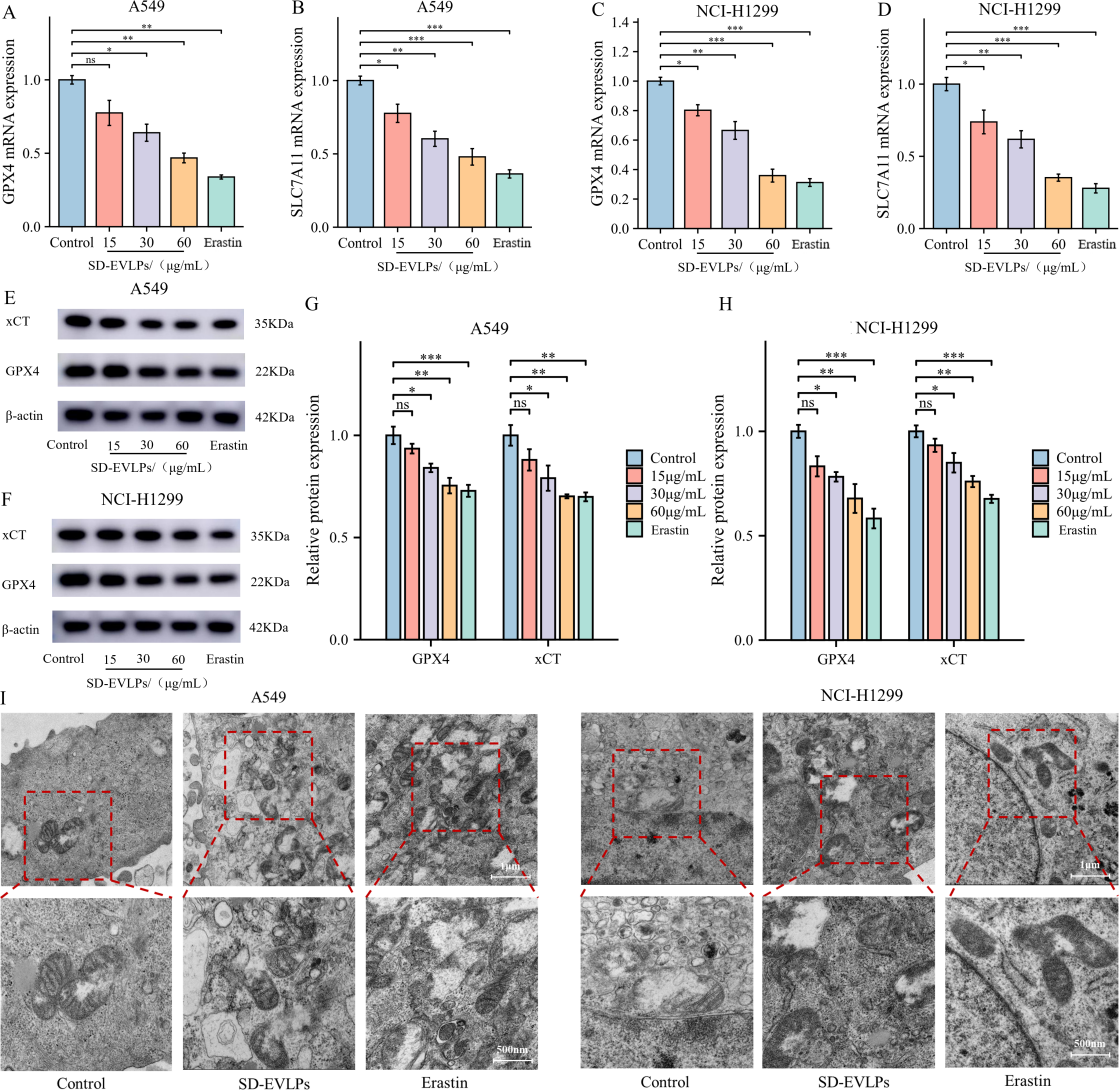
RT-qPCR and Western blot analysis of ferroptosis-related gene expression in lung cancer cells **(A, B)** RT-qPCR analysis of *GPX4* and *SLC7A11* mRNA expression in A549 cells; **(C, D)** RT-qPCR analysis of *GPX4* and *SLC7A11* mRNA expression in NCI-H1299 cells; **(E–H)** Western blot analysis of GPX4 and xCT protein expression in lung cancer cells; **(I)** TEM assessment of mitochondrial ultrastructure. Data are presented as the mean ± SEM, n = 3 independent experiments. Compared with the control group, *P < 0.05, **P < 0.01, and ***P < 0.001.

Ferroptosis is characterized by distinct mitochondrial morphological alterations, including reduced or lost cristae and increased membrane density. To determine whether SD-EVLPs treatment induced mitochondrial ultrastructural alterations consistent with ferroptosis, TEM was employed to examine mitochondrial ultrastructure in A549 and NCI-H1299 cells. Control cells displayed normal mitochondrial morphology, including intact double membranes, preserved cristae, and appropriate size and density. In contrast, cells treated with SD-EVLPs or erastin showed prominent mitochondrial damage after 48 h, exhibiting typical ferroptosis-associated pathological features such as shrunken mitochondrial volume, increased membrane density, focal outer membrane rupture, and blurred or absent cristae structures. These ultrastructural abnormalities were consistent with ferroptosis-associated mitochondrial injury, suggesting that mitochondrial dysfunction may contribute to the ferroptosis-related response observed after SD-EVLP treatment (**Figure 9**).

#### 3.4.2 SD-EVLPs modulate the FABP4/PPARG/GPX4-associated pathway in lung cancer cells

**Figure 10:**
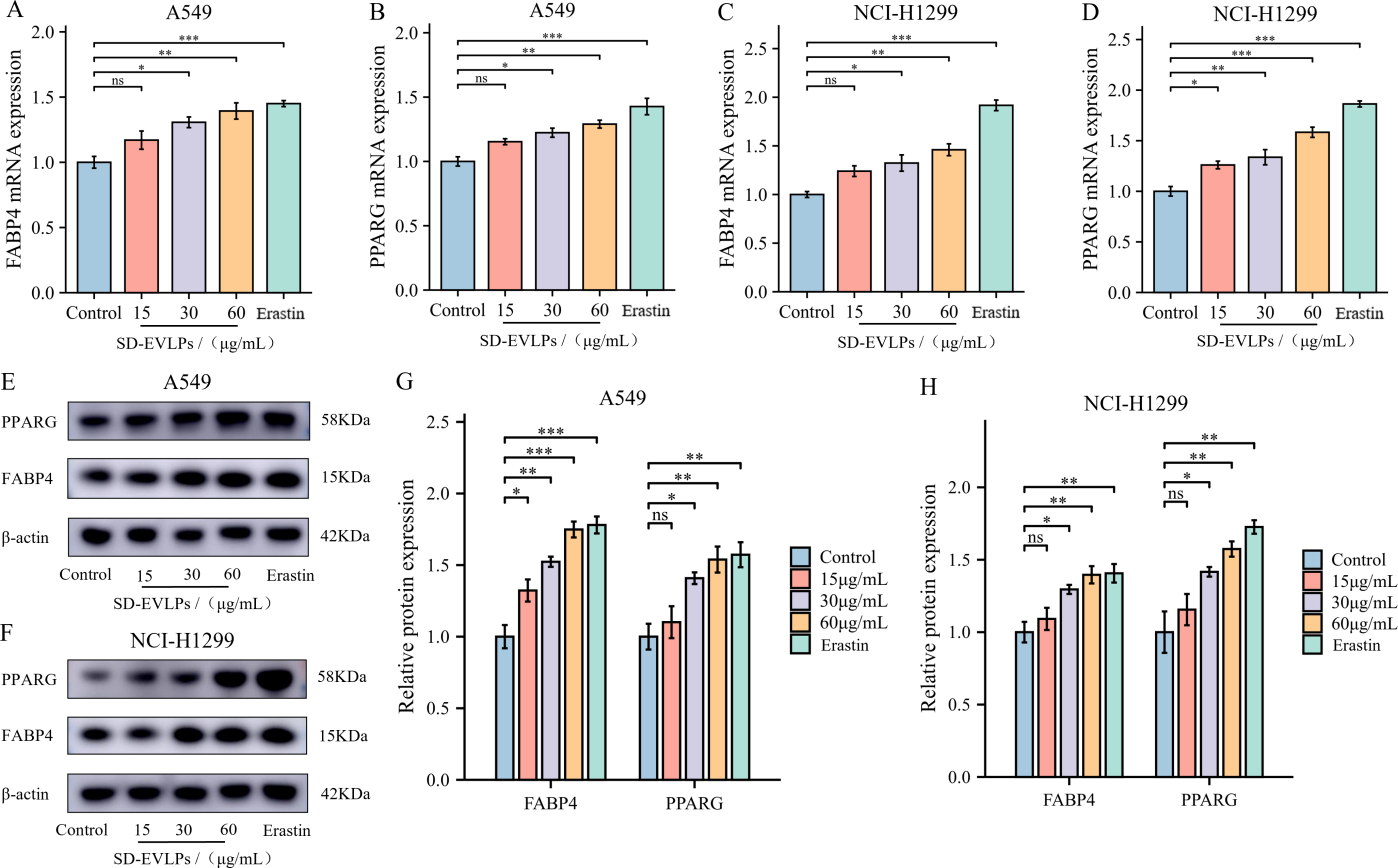
SD-EVLPs modulate the FABP4/PPARG/GPX4-associated pathway in lung cancer cells **(A–D)** RT-qPCR analysis of *FABP4* and *PPARG* mRNA expression in A549 and NCI-H1299 cells; **(E–H)** Western blot analysis of FABP4 and PPARG protein expression in A549 and NCI-H1299 cells. Data are presented as the mean ± SEM, n = 3 independent experiments. Compared with the control group, *P < 0.05, **P < 0.01, and ***P < 0.001.

KEGG enrichment analysis initially suggested that the molecular mechanism underlying SD-EVLPs-promoted ferroptosis may involve modulation of the PPAR signaling pathway, in which the key effector molecules *FABP4* and *PPARG* appear to play central roles. To elucidate this mechanism in greater depth, RT-qPCR and Western blot analyses were performed to systematically evaluate the regulatory effects of SD-EVLPs on *FABP4* and *PPARG* at both the transcriptional and translational levels. The results showed that SD-EVLPs exerted a dose-dependent positive regulatory effect on *FABP4* and *PPARG* expression in both A549 and NCI-H1299 lung cancer cells. Medium and high concentrations of SD-EVLPs (30 and 60 μg/mL), as well as erastin (20 μmol/L), significantly upregulated the mRNA levels of *FABP4* and *PPARG* and the protein levels of FABP4 and PPARG compared with the control group (*P* < 0.05, *P* < 0.01, *P* < 0.001). In addition, the upregulating effect of high-concentration SD-EVLPs was comparable to that of erastin. Taken together, these findings suggest that SD-EVLP treatment is associated with modulation of the FABP4/PPARG/GPX4-associated pathway. SD-EVLPs increase the expression of FABP4 and PPARG, while simultaneously suppressing GPX4 and xCT expression, thereby disrupting cellular redox homeostasis and promoting lipid peroxidation (**Figure 10**).

### 3.5 Effect of SD-EVLPs on subcutaneous xenografts of lung cancer in nude mice

We successfully established a subcutaneous xenograft model of A549 cells in BALB/c nude mice and administered drug interventions for 14 days. The *in vivo* results showed that, compared with the control group, tumor growth was markedly slowed in the SD-EVLPs-L, SD-EVLPs-H, and cisplatin groups, with significantly reduced tumor volumes (*P* < 0.01, *P* < 0.001). Among the three treatment groups, mice in the cisplatin group exhibited a more pronounced decrease in body weight, whereas body weight in the SD-EVLPs groups remained generally stable with only a mild decrease.

H&E staining revealed that tumor tissues in the control group exhibited densely arranged cells with vigorous proliferation, clear cellular boundaries, deeply stained nuclei, hypertrophic morphology, abundant cytoplasm, and frequent mitotic figures and multinucleated cells. In contrast, in both the SD-EVLPs and cisplatin groups, we observed dispersed tumor cells, reduced cellular density, cell rupture, cytoplasmic leakage, numerous vacuolar structures, and nuclear pyknosis, fragmentation, and dissolution, accompanied by extensive necrosis. Ki-67 is a classical proliferation-related protein that maintains chromosome separation during cell division, and its expression level reflects cellular proliferative activity. Higher Ki-67 expression indicates a larger proportion of cells in the division phase and more active proliferation. Ki-67 is widely used as a tumor marker in pathological research and shows minimal expression in certain normal tissues (22). Using IHC staining, we found that Ki-67 expression in tumor tissues was significantly reduced in the SD-EVLPs and cisplatin groups compared with the control group (*P* < 0.01, *P* < 0.001), indicating that both SD-EVLPs and cisplatin markedly inhibit lung cancer tumor growth.

**Figure 11:**
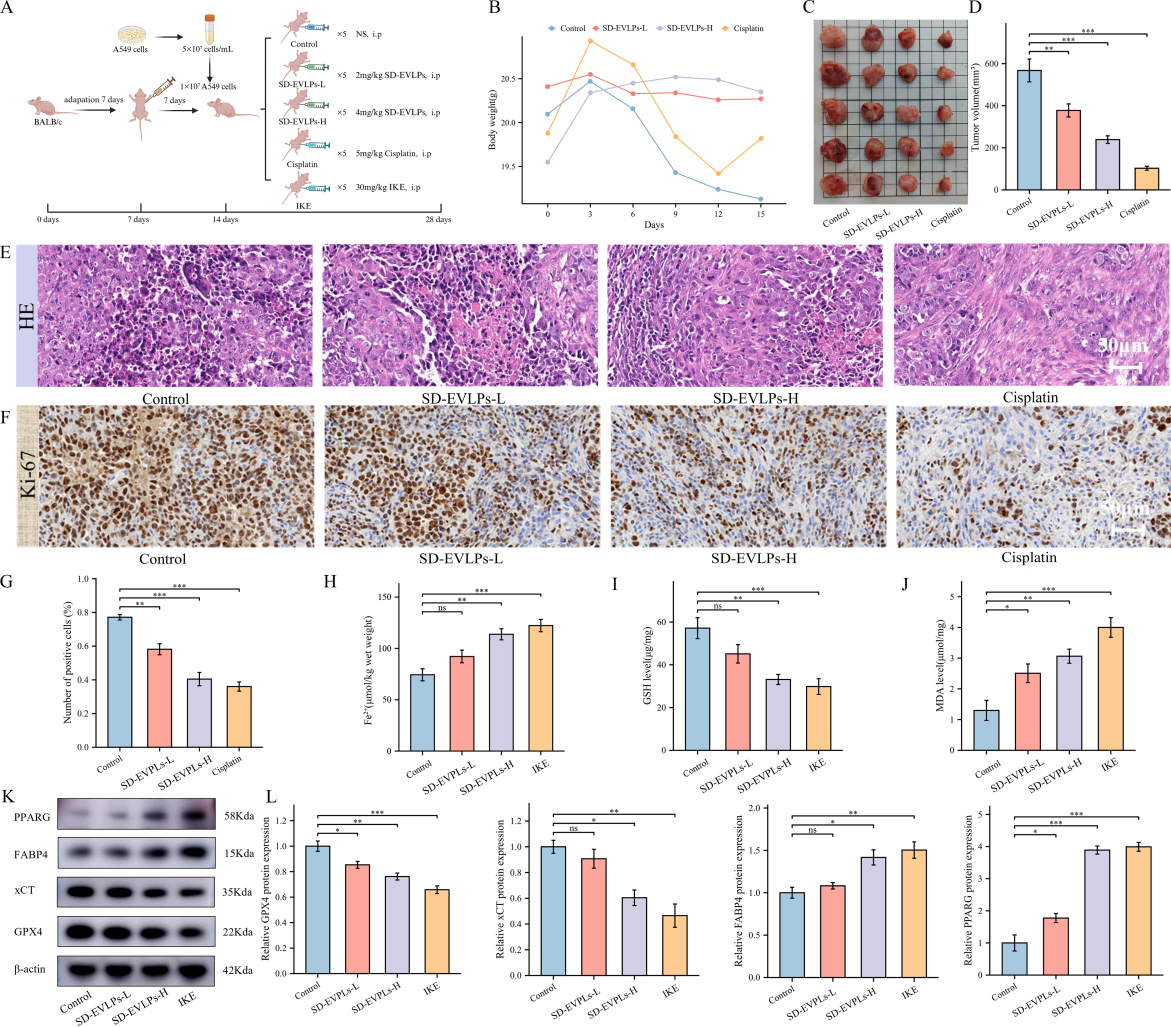
SD-EVLPs are associated with ferroptosis-related changes and modulation of the FABP4/PPARG/GPX4 pathway *in vivo* **(A)** Preparation of the subcutaneous xenograft tumor model in nude mice and the drug intervention method and timeline; **(B)** Changes in body weight of nude mice; **(C)** Tumor tissue samples; **(D)** Changes in tumor volume; **(E)** Histopathological changes in tumor tissues assessed by H&E staining; **(F, G)** Expression of Ki-67 in tumor tissues detected by IHC; **(H–J)** Measurement of Fe²^+^, GSH, and MDA content in tumor tissues; **(K, L)** Protein expression of GPX4, xCT, FABP4, and PPARG in tumor tissues detected by Western blot. Data are presented as the mean ± SEM, n = 5 mice per group. Compared with the control group, *P < 0.05, **P < 0.01, and ***P < 0.001.

To further explore the correlation between SD-EVLP-mediated inhibition of tumor growth *in vivo* and the promotion of tumor ferroptosis as well as pathway proteins, we measured Fe²^+^, GSH, and MDA levels in tumor tissues using colorimetric assays, and assessed the expression of ferroptosis-related proteins (GPX4 and xCT) and key pathway proteins (FABP4 and PPARG) using Western blotting. The results showed that, compared with the control group, Fe²^+^ and MDA levels were significantly increased in the SD-EVLPs-H and IKE groups, whereas GSH levels were significantly decreased (*P* < 0.01, *P* < 0.001). In addition, the expression of GPX4 and xCT was significantly downregulated (*P* < 0.05, *P* < 0.01, *P* < 0.001), while the expression of FABP4 and PPARG was significantly upregulated (*P* < 0.05, *P* < 0.01, *P* < 0.001). Based on these findings, we propose that the inhibitory effect of SD-EVLPs on lung cancer growth *in vivo* is associated with ferroptosis-related changes potentially involving modulation of the FABP4/PPARG/GPX4-associated pathway (**Figure 11**).

### 3.6 Biological safety assessment of SD-EVLPs

The liver and kidneys are essential organs involved in immune regulation, metabolism, and detoxification, and their functional integrity directly influences the therapeutic applicability of drugs. ALT and AST serve as sensitive indicators of liver injury, while BUN and CREA are key parameters for evaluating renal function. In this study, no significant differences were observed in the levels of ALT, AST, BUN, or CREA between the SD-EVLPs-treated mice and the control group (*P* > 0.05), indicating that SD-EVLPs did not cause detectable hepatic or renal dysfunction.

**Figure 12:**
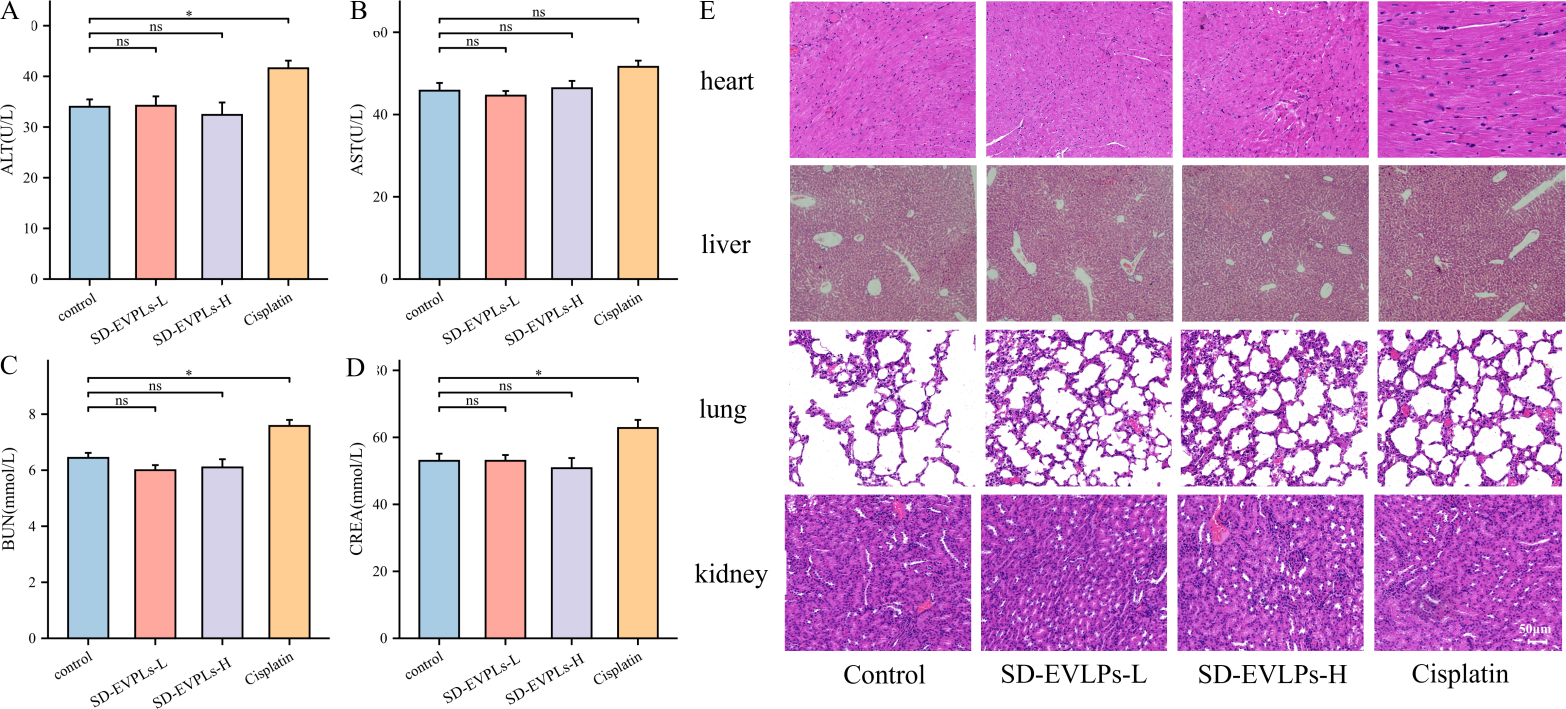
Biological safety of SD-EVLPs **(A)** Measurement of ALT levels; **(B)** Measurement of AST levels; **(C)** Measurement of BUN levels; **(D)** Measurement of CREA levels; **(E)** H&E staining of heart, liver, lung, and kidney tissues. Data are presented as the mean ± SEM, n = 5 mice per group. Compared with the control group, *P < 0.05.

To further assess the biological safety of SD-EVLPs, H&E staining was performed on the heart, liver, lungs, and kidneys of nude mice. The results showed that the tissue architecture and cellular morphology of all examined organs remained normal in both the control and SD-EVLPs groups, with no evidence of tumor metastasis. These findings suggest that SD-EVLPs exhibit favorable biological safety (**Figure 12**).

## 4 Discussion

This study systematically investigated the anti–lung cancer activity of SD-EVLPs and their association with ferroptosis-related changes. We successfully isolated SD-EVLPs from *Selaginella doederleinii* and characterized their morphology and size distribution, while LC–MS profiling indicated a multicomponent material basis. Integrated bioinformatics analyses further suggested that the FABP4/PPARG/GPX4 regulatory axis enriched in the PPAR signaling pathway may represent a candidate mechanistic direction. *In vitro* and *in vivo* experiments corroborated that SD-EVLPs suppressed Non-Small Cell Lung Cancer (NSCLC) cell proliferation, migration, and invasion, as well as subcutaneous xenograft growth. Importantly, the molecular alterations observed experimentally were concordant with the bioinformatically prioritized PPAR-associated signatures, suggesting that the FABP4/PPARG/GPX4-associated pathway may represent a candidate mechanistic node of interest in the SD-EVLP response and supporting an association between the antitumor effects of SD-EVLPs and ferroptosis-related changes in lung cancer cells. Collectively, these findings provide an interpretable evidence chain supporting the integration of bioactive medicinal plants with natural nanodelivery platforms and indicate the preclinical translational promise of SD-EVLPs.

Lung cancer remains a leading cause of cancer incidence and mortality. Although surgery, radiotherapy/chemotherapy, targeted therapy, and immunotherapy have substantially advanced, treatment-related toxicity, drug resistance, and limited benefit in eligible populations continue to constrain outcome improvement. As a traditional Chinese medicinal herb with documented antitumor activity, *Selaginella doederleinii* has attracted growing interest for lung cancer-related research. Wang et al. reported that *Selaginella doederleinii* extracts promote apoptosis and inhibit proliferation and migration of A549 and PC9 cells (23). Using network pharmacology and proteomics, Xu et al. suggested that bioactive biflavonoids may suppress NSCLC through multicomponent, multitarget regulation involving MAPK and PI3K/AKT signaling networks, with putative targets including EGFR, AKT, and MEK (24). In a Lewis lung cancer model, Yao et al. observed that total biflavonoid extracts inhibited tumor growth accompanied by reduced Ki67 expression and decreased microvessel density (25).

With the rapid development of PDEVs research, accumulating evidence indicates that PDEVs can deliver bioactive lipids, proteins, nucleic acids, and small molecules, thereby participating in immunomodulation, inflammatory microenvironment remodeling, and drug delivery, with emerging potential in antitumor therapy (26, 27). Compared with synthetic nanoparticles, PDEVs show advantages in biocompatibility, low immunogenicity, gastrointestinal stability, cellular uptake, and tissue delivery (28, 29). Previous studies reported that ginseng-derived PDEVs can cross the blood–brain barrier and modulate the tumor immune microenvironment to suppress glioma growth (18), and may enhance the efficacy of immune checkpoint inhibitors by reprogramming tumor-associated macrophages and related mechanisms (30). In addition, mulberry leaf–derived PDEVs exhibit liver tropism, regulate gut microbiota, and suppress hepatocellular carcinoma growth (31). Grapefruit-derived nanovesicles have also been used to deliver miR-17 and inhibit brain tumor progression in mice (32). On this basis, we prepared SD-EVLPs to leverage PDEV-like features to enhance the antitumor advantages of *Selaginella doederleinii.* SD-EVLPs were isolated using ultracentrifugation and sucrose density gradient centrifugation and validated by TEM, NTA, and BCA-based protein quantification, supporting a relatively stable nanovesicle-like profile. LC–MS identified 93 chemical constituents in SD-EVLPs, and further screening prioritized 25 candidate bioactive components, suggesting that the biological effects of SD-EVLPs may arise from multicomponent cooperation. Together, the SD-EVLP preparation and characterization workflow provides a methodological reference for upgrading fresh-herb preservation strategies and standardizing PDEV extraction, and establishes a foundation for subsequent functional and mechanistic studies.

Ferroptosis is a regulated form of cell death characterized by iron-dependent lipid peroxide accumulation coupled with failure of antioxidant defense systems (2), and it has attracted sustained attention in cancer therapy and resistance reversal (33). Lung cancer cells frequently exhibit dysregulated iron metabolism and redox imbalance, which can confer vulnerability to ferroptosis induction under specific conditions (34). Prior studies indicate that Fe²^+^-loaded iron-based nanoparticles can activate Fenton chemistry in NSCLC cells, inducing ferroptosis and suppressing proliferation and migration, with potential to improve chemotherapy resistance (35, 36). The small-molecule ferroptosis inducer PRLX93936 has been reported to downregulate xCT, disrupt the GSH–GPX4 antioxidant axis, and synergize with cisplatin to enhance chemosensitivity in NSCLC (37). Cisplatin itself can deplete intracellular GSH and inhibit GPX4 activity in A549 cells, serving as a key trigger of ferroptosis (38). These findings support ferroptosis regulation as a promising entry point to improve lung cancer treatment and overcome resistance.

We next applied integrated bioinformatics, molecular docking, and MD simulations to interrogate key targets and pathways potentially involved in SD-EVLP–mediated ferroptosis in lung cancer. By intersecting predicted targets of SD-EVLP constituents, ferroptosis-related genes, and lung cancer differentially expressed genes, we identified 17 candidate targets, including FABP4, PPARG, and IL6, and further prioritized them based on differential expression, prognostic association, and pathway enrichment. Given the enrichment evidence and prognostic relevance, the mechanistic discussion was focused on the FABP4/PPARG/GPX4 axis enriched in the PPAR signaling pathway. Notably, the PPAR pathway is not considered a canonical “core axis” of ferroptosis. However, it can reshape transcriptional programs governing fatty-acid uptake, transport, and oxidation (39), thereby influencing membrane phospholipid composition and the oxidative burden threshold, which may act as an upstream layer modulating ferroptosis susceptibility (40, 41). Docking and MD analyses supported strong binding affinity and stable binding conformations between multiple SD-EVLP constituents and FABP4, PPARG, GPX4, and xCT. Consistently, experimental validation showed that SD-EVLPs inhibited lung cancer progression and were accompanied by ferroptosis-related changes, with modulation of the PPAR-enriched FABP4/PPARG/GPX4-associated pathway observed in parallel. Beyond FABP4 and PPARG, other candidate targets such as IL6 and AURKA may also contribute to antitumor effects, with IL6 more plausibly related to inflammatory signaling and immune microenvironment regulation and AURKA linked to cell-cycle and proliferative control. In this study, we prioritized the ferroptosis-relevant FABP4/PPARG/GPX4 axis while retaining openness to additional targets and pathways as important directions for future work. Mechanistically, FABP4, a fatty acid–binding protein family member, participates in intracellular fatty-acid transport and metabolism, promotes lipid accumulation, and can increase the abundance of polyunsaturated fatty acids (PUFAs) within membrane phospholipids, thereby providing substrates for lipid peroxidation (42). FABP4 has also been implicated in ferroptosis-related regulation through effects on lipid metabolism, oxidative stress, and mitochondrial function (42), and aberrant FABP4 expression has been associated with tumor initiation and progression across cancer types (43). In lung cancer, reduced FABP4 expression correlates with higher malignancy and poorer prognosis. Consistently, FABP4 is reported to be downregulated in lung cancer, and SIRT5 may promote NSCLC progression by reducing FABP4 acetylation (44). Moreover, Src inhibition has been shown to relieve suppression of PPARγ transcriptional activity and upregulate FABP4, accompanied by reduced lipid droplets and increased intracellular ROS, ultimately attenuating tumor growth (45). These observations support the view that FABP4 participates in lipid metabolic reprogramming and may influence ferroptosis susceptibility through substrate availability and redox context.

PPARG (PPARγ) is a nuclear receptor transcription factor that serves as a key node integrating lipid metabolism and inflammatory signaling. Its dysregulated expression has been documented across multiple cancers and is associated with tumor proliferation, apoptosis, and invasion (46–48). Multi-cohort integrative analyses indicate that PPARG is significantly downregulated in lung adenocarcinoma and correlates with unfavorable prognosis, potentially linked to aberrant activation of molecular networks governing proliferation and metastasis (49, 50). Studies have also shown that PPARG agonists combined with radiotherapy/chemotherapy can synergistically inhibit NSCLC cell growth and enhance sensitivity to radiotherapy/chemotherapy (41). Activation of PPARG may inhibit the initiation and progression of lung squamous cell carcinoma by modulating upstream regulators and downstream marker genes, supporting PPARG as a therapeutic target in lung squamous cell carcinoma (51). At the transcriptional level, PPARG drives the expression of genes involved in fatty-acid uptake, transport, and lipid metabolism, and FABP4 is widely recognized as a classic PPARG target gene; PPARγ activation can upregulate FABP4 via PPAR response elements in its promoter (52). FABP4 can also bind long-chain fatty acids and undergo nucleo–cytoplasmic shuttling, which has been proposed to affect ligand availability to nuclear PPARγ and enhance PPARγ-dependent transcriptional output (53, 54). In this context, concordant changes in FABP4 and PPARG are consistent with coordinated regulation of fatty-acid mobilization/partitioning and PPARG-driven lipid-metabolic transcription.

**Figure 13:**
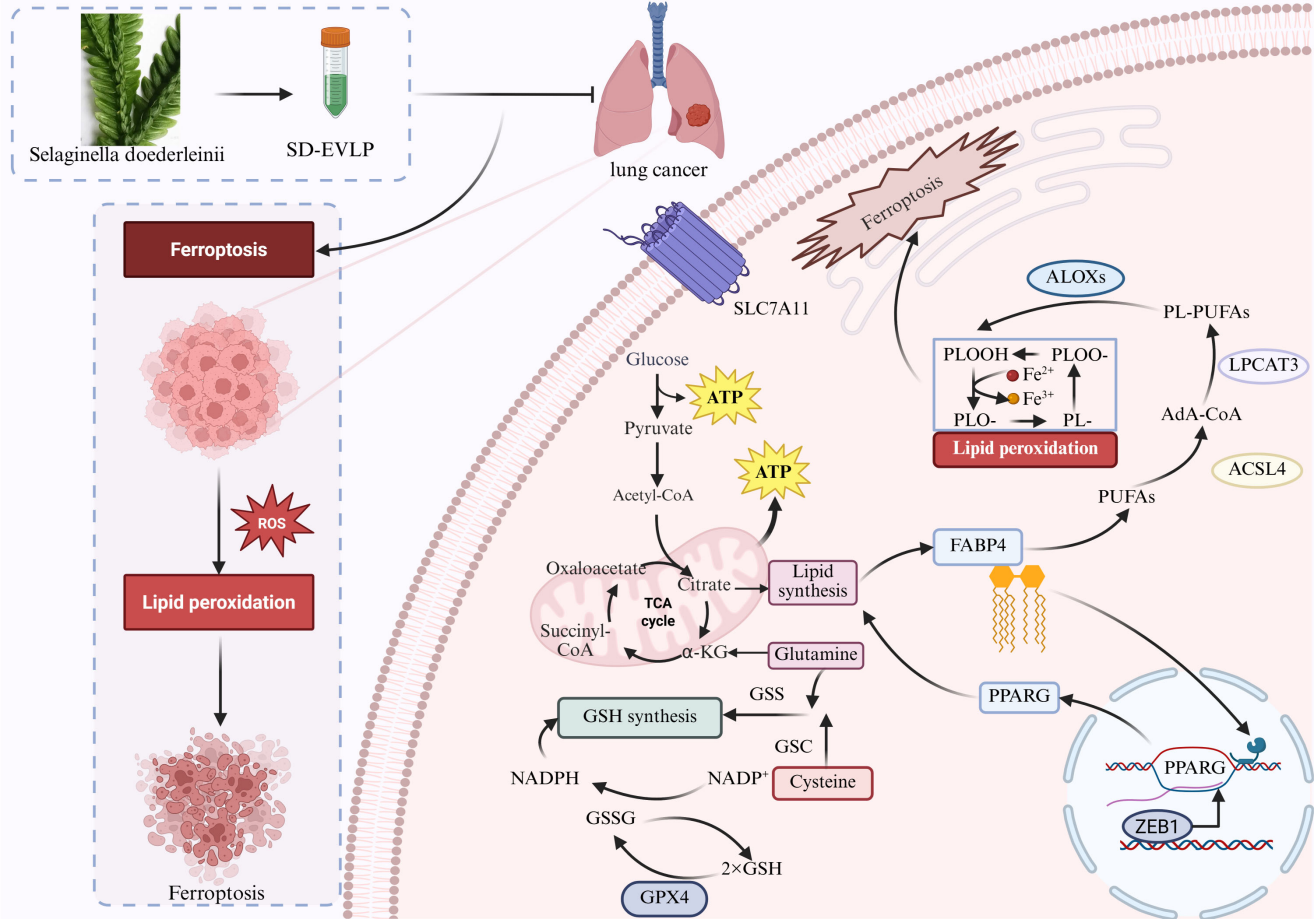
Mechanism diagram.

With respect to ferroptosis, such remodeling of lipid metabolic programs can increase PUFA-containing phospholipid substrates and elevate lipid peroxidation burden. When cystine import mediated by xCT, GSH synthesis, and GPX4-dependent reduction/clearance of phospholipid hydroperoxides are compromised, lipid peroxides can accumulate persistently and trigger ferroptosis (55). Consistent with this framework, during EMT in lung cancer, ZEB1 has been reported to directly activate PPARG, enhance synthesis of PUFA-containing phospholipids, and suppress GPX4 antioxidant function, thereby increasing ferroptosis sensitivity in mesenchymal-like cells (40). In addition, GPX4 inhibitors have been reported to impair dendritic cell maturation and antitumor function by activating PPARG-dependent ferroptosis (56). PPARG agonists such as pioglitazone have also been suggested to induce metabolic reprogramming, suppress SLC7A11 expression, and ultimately lead to GSH depletion and GPX4 inactivation (57). Together with our findings, these lines of evidence support the working model that SD-EVLPs may modulate FABP4/PPARG-associated lipid-metabolic programs and concurrently impact the GPX4- and xCT-centered antioxidant defense, thereby facilitating lipid peroxide accumulation and ferroptosis-related changes that accompany suppression of lung cancer progression (**Figure 13**).

Several limitations should be acknowledged. First, only two NSCLC cell lines and a subcutaneous xenograft model were used; the efficacy and pharmacodynamic features of SD-EVLPs in orthotopic or metastatic lung cancer models require further evaluation. Second, although multi-omics prediction and experimental results collectively support the FABP4/PPARG/GPX4 axis as a potentially important regulatory node, direct genetic or pharmacological perturbation of these targets was not performed, and causality remains to be established. Nonetheless, the dose-dependent concordance between key node alterations, ferroptosis markers, and antitumor phenotypes supports a strong association; future studies using knockdown/overexpression or pharmacological perturbations are needed to confirm causal roles. Third, as PDEV bioactivity often arises from multicomponent cooperation, the relative contribution of individual SD-EVLP constituents to ferroptosis promotion remains unclear and should be addressed through fractionation, add-back, and functional validation. Overall, these limitations do not undermine the principal observations or the interpretive framework of this study. Future work will further focus on pharmacokinetics, optimization of administration routes, and formulation development to advance the translational potential of SD-EVLPs.

## 5 Conclusion

In this study, SD-EVLPs were successfully isolated and their bioactive constituents were identified. By integrating bioinformatics analyses with *in vitro* and *in vivo* experiments, we found that SD-EVLPs suppressed lung cancer progression and were associated with ferroptosis-related changes. These effects may involve modulation of the FABP4/PPARG/GPX4-associated pathway within the PPAR signaling network. Collectively, these findings highlight SD-EVLPs as a promising natural nano-therapeutic candidate and provide a mechanistically informed framework for understanding how PDEVs may interface with modern molecular pathways in cancer therapy.

## Data Availability

The datasets presented in this study can be found in online repositories. The names of the repository/repositories and accession number(s) can be found in the article/supplementary material.
